# Alcohol Misuse in Older Women: A Scoping Review of Correlates, Consequences, Treatment, and Prevention

**DOI:** 10.35946/arcr.v46.1.02

**Published:** 2026-05-14

**Authors:** Cathryn Glanton Holzhauer, Madeleine Stein, Rachel Rosen, Hannah Grigorian, Marissa Iverson, Elizabeth E. Epstein

**Affiliations:** 1 Department of Psychiatry, University of Connecticut School of Medicine, Farmington, Connecticut,; 2 Department of Psychiatry, University of Massachusetts Medical School, Worcester, Massachusetts,; 3 Narrows Institute for Biomedical Research & Education Inc., Brooklyn, New York; 4 Department of Psychiatry, Massachusetts General Hospital, Boston, Massachusetts; 5 Mental Illness Research, Education and Clinical Center, VA Bedford Healthcare System, Bedford, Massachusetts; 6 UConn Health Sciences Library, Farmington, Connecticut

**Keywords:** alcohol, women, female, aging, older, treatment, senior, substance

## Abstract

**BACKGROUND:**

Alcohol use and alcohol use disorder (AUD) among older women (defined as age 50 and over) have increased substantially in recent years. Compared to men and younger women, older women negotiate biopsychosocial transitions that put them at greater risk of alcohol-related health conditions as they age and are less reliably screened and treated for heavy alcohol use and AUD.

**OBJECTIVES:**

This scoping review represents a critical evaluation of research methodology and findings since 2004 on biopsychosocial correlates and consequences of alcohol misuse (i.e., heavy alcohol use, defined as four or more drinks on any day or eight or more drinks per week; and/or AUD) among older women. These findings, with a focus on their implications for treatment and prevention strategies among this at-risk population, are discussed. The goal of the review is to summarize current research as well as treatment and prevention options available for older women and to identify gaps in the literature and potential for future research.

**ELIGIBILITY CRITERIA:**

2,579 research articles were reviewed for inclusion in the paper. Inclusion criteria required that a study’s findings were relevant to the review’s objectives, with analyses and results that had clinically relevant findings for older women with heavy alcohol use and/or AUD.

**SOURCES OF EVIDENCE:**

Authors searched PubMed, Embase, and PsycInfo for articles published between January 1, 1994, and August 5, 2024.

**CHARTING METHODS:**

Two authors developed the template for data extraction, and four authors charted data. Weekly meetings were used to establish, monitor, and maintain calibration among authors.

**RESULTS:**

127 articles met inclusion/exclusion criteria and were included in the final extraction and results summary. Evidence-based information is presented on (1) clinical presentation of older women with alcohol misuse; (2) biological correlates of alcohol use in older age; (3) psychosocial correlates and consequences, such as mood disorders, social networks, and other substance use; (4) increased risk of health conditions, such as breast cancer and depression; and (5) current state of treatment and prevention needs and efforts.

**CONCLUSIONS:**

The research reviewed here indicates a need for further age- and sex-specific research in the field of alcohol use and AUD. After age 50, men and women continue to differ in important ways in relation to the correlates, consequences, and treatment of alcohol misuse. The current literature includes significant methodological limitations and inconsistencies. Research with samples of older women who drink at heavy levels may be particularly helpful in addressing these limitations. Existing prevention and treatment interventions may be good options for older women, but more research is needed.

**KEY TAKEAWAYS**

- Documented sex and age differences in the correlates and consequences of alcohol misuse, increasing rates of alcohol use disorder (AUD) among older women, and the accelerated negative impact of AUD on women’s health all underscore the importance of further research with older women.
- After age 50, men and women continue to differ in important ways in terms of biopsychosocial correlates and consequences of alcohol use (e.g., co-occurring conditions, social networks, physical conditions secondary to alcohol use, treatment needs).
- Primary prevention efforts may be key for older women, including awareness information campaigns tailored to lifespan phase.
- The current literature on older women includes significant methodological limitations and inconsistencies.
- More research that targets recruitment of older women with heavy alcohol use and/or AUD could be especially beneficial.

## Rationale

The United States’ population of men and women who are in older adulthood is growing. Due to the largest generational cohort to date—the baby boomers—entering late adulthood, the number of Americans age 65 and older is projected to increase to 82 million by 2050 (a 47% increase since 2022).^1^ Born between 1946 and 1964, those in the baby boomer generation are, as of 2025, between 61 and 79 years old. In parallel with the increase in the population of older adults, there has also been a trajectory of sex convergence over the last 25 years in the rates of alcohol use disorder (AUD) and other substance use disorders,^2^,^3^ especially among older adults.^4^ Women age 50 and older are consuming more alcohol,^5^ developing AUD at higher rates,^4^ and experiencing greater increases in alcohol-related mortality^6^ and other alcohol-related problems^7^ compared to previous cohorts. AUD among older women has been identified as an emerging public health issue,^4^,^6^ especially as alcohol use among women has been increasing and rates among men have either remained stable or decreased.^3^ The 2024 National Survey on Drug Use and Health found that 8% of women (10.7 million) age 21 and older had AUD in the past year, compared to 13% of men (15.8 million) age 21 and older.^8^ Among women age 50 and older, the past-year prevalence of AUD increased by 85% between 2005 and 2013.^4^ Moreover, in women age 65 and older, past-month drinking increased 27% (up to 38%) from 2002 to 2018, with rates of alcohol use increasing among those age 65 and older in general.^9^ Data on changes in alcohol use among adults over age 55 during the COVID-19 pandemic also indicated more recent increases in rates of alcohol use among this population.^9^

The reasons for increases in alcohol use and misuse (defined by the National Institute on Alcohol Abuse and Alcoholism [NIAAA] as drinking in a manner, situation, amount, or frequency that could cause harm to the person who is engaging in drinking or to those around them)^10^ among older adults have yet to be fully elucidated. Hypothesized explanations include the following:

- Changing social roles—for example, historically, expectations for women to conduct all child care and domestic labor precluded engagement in social opportunities for drinking among women, but not men;
- Changing social views on religion—in the United States, many religious groups have sanctions on alcohol use, especially among women, and religious involvement has decreased or changed over the years for many individuals;^11^
- Marketing and advertising that targets women, including inaccurate messaging about purported health benefits of “moderate drinking”;^12^
- Longer and healthier lifespans— women now remain healthier for more years and therefore may be more likely to drink alcohol; historically, greater rates of illness or chronic conditions among women earlier in life led to less drinking so as to not exacerbate health conditions (see below about the “sick quitter effect”);^12^
- Socioeconomic risk factors, such as financial strain and decreased income in older adults.^13^

These factors are likely to interact with biological and psychological risk factors to increase alcohol use in older women.^12^ Additionally, the baby boomer generation may have always consumed alcohol at higher rates, as suggested by the fact that the highest per-capita alcohol consumption in the United States occurred in the early 1980s, when most baby boomers were young adults. Epidemiological research suggests that this cohort has maintained these rates of alcohol consumption over the years.^9^ However, despite extensive evidence of increasing rates of alcohol use and AUD in older women, there is a paucity of research on, and treatments or prevention strategies for, this population.

Since 1993, the National Institutes of Health (NIH) has required biomedical researchers to include female participants in clinical research and to design NIH Phase III clinical trials that permit analysis of sex differences.^14^ As a result, several evidence- and treatment-needs-based female-specific AUD treatments and prevention strategies are emerging.^15^–^17^ More recently, NIH revised its policy and guidelines on the inclusion of research participants across the lifespan to include adults of all ages, including older adults.^18^ Thus, research on treatment, prevention strategies, and needs among older women with alcohol misuse is expected to accumulate more rapidly going forward. As described in more detail below, research on alcohol use and AUD over the past 25 years, for the most part, has included both male and female participants; however, most studies have not analyzed data or presented results by stratified sex and/or age, precluding interpretation of results specifically for older women. Studies presented in the current scoping review are notable exceptions in the literature since 2004.

## Notes on Terminology and Definitions

For the current review, the term “older” women refers to individuals age 50 and older. Lifespan development theory generally differentiates chronological ages of early adulthood (ages 18 to 44), middle adulthood (ages 45 to 55), and late adulthood (age 56 and older); however, actual ages used in various studies to reflect these lifespan phases are inconsistent.^19^ Thus, onset of “late” or “older” adulthood can range from age 50 and older to age 65 and older.^19^ This review defines “older woman” as age 50 and older for several reasons. Evidence of sex convergence in prevalence rates of AUD and binge drinking (defined by NIAAA as a drinking pattern that brings blood alcohol concentrations to 0.08% or higher, which typically corresponds to five or more drinks for men and four or more drinks for women in about 2 hours)^20^ in older adults includes those age 50 and older.^3^–^5^ As described below in the Methods section, several “sentinel” articles were identified to help generate the search criteria. Through that process, it was evident that many of the articles that had important implications for older women used samples of women with a cutoff of age 50 and older. Relatedly, given that older women remain an under-researched subpopulation, age 50 and older was chosen to cast a “wide net” (opposed to using a cutoff of age 60 or 65) and provide the best chances of retaining quality research. This “wide-net” approach also allowed for identification of longitudinal research that followed women from age 50 into later years.

A major methodological problem across articles in this literature was inconsistency and/or misnomers in definitions of alcohol consumption, which could lead to misinterpretation of results and/or inaccurate conclusions. For example, many studies used the phrase “moderate drinking” to characterize some amount of alcohol intake that was idiosyncratic to each article. For this scoping review, to avoid inaccuracies in interpreting and reporting results, drinking patterns (including the word “moderate” and other uninterpretable language) in each article were converted to align with NIAAA definitions.^20^ For example, NIAAA defines heavy drinking as consuming five or more drinks on any day or 15 or more drinks per week for men, and four or more drinks on any day or eight or more drinks per week for women. When the term “binge drinking” is used in this paper, it refers to consuming four or more drinks on one occasion for women, unless otherwise stated (some papers used non–sex-tailored definitions as their binge drinking measures, preventing translation of the findings). For continuous measures of alcohol consumption, the results were translated and presented as needed in U.S. standard drinks (14 grams of pure alcohol). For example, because a “unit” of alcohol in the United Kingdom refers to 10 grams of pure alcohol, findings based on number of units were converted to U.S. standard drinks; therefore, whenever the term “drink(s)” is used in the manuscript, it is referring to U.S. standard drinks.

The studies included in this review used a variety of measures to assess alcohol use and alcohol-related problems in older women. A summary of these measures, the constructs measured, and the scoring and interpretation of the items are outlined in Table 1.

## Age-Neutral Literature on Women With Alcohol Misuse

The existing age-neutral scientific literature provides information on aspects of etiology, mortality, triggers, metabolism, risk, relapse antecedents, clinical presentation, and course of alcohol misuse and AUD among women generally (not specifically older women) compared to men.^17^,^30^ At all ages, women metabolize alcohol less efficiently than men do and may incur worse harm and more negative consequences despite using less alcohol or other drugs than men do (a phenomenon referred to as the “telescoping effect”). At any age, women who misuse alcohol are at heightened risk for adverse medical consequences, including heart disease, liver inflammation, liver disease, cardiovascular disease, brain damage and cognitive deficits, and cancers (e.g., breast, colorectal), compared to men who misuse alcohol.^17^,^20^,^31^ In terms of AUD recovery, mechanisms of change also may differ by sex.^17^ Although this issue is under-researched, AUD treatments appear to be equally effective for men and women.^17^ As outlined below, much less is known about older women specifically.

## Aging and Menopause-Related Factors in Alcohol Research

Unique issues experienced by women with AUD or heavy drinking that are revealed in the wider age- and sex-neutral literature remain relevant to older women, because samples in that wider literature often include women over age 50. This review will discuss issues that are more likely to arise with age, such as psychosocial changes and medical sequelae of female aging, with a focus on how they might guide development of treatment and prevention strategies for older women. However, as illustrated below, there is a paucity of literature on many of these age-related factors among older women. Although age-neutral literature would suggest, for instance, that widowhood and isolation may be central to risk and maintenance of AUD in older women (i.e., based on research that has shown the importance of partnership, social support, and social networks in determining women’s alcohol use^17^,^31^), not enough research has addressed this topic. Consequently, age-related social, psychological, and biological changes that would be hypothesized to increase risk for alcohol misuse and AUD in older age represent critical areas for continued research.

As mentioned, at all ages, women start to experience problems related to their alcohol use sooner and at lower drinking levels than men do^32^ and are more vulnerable to negative effects of alcohol consumption.^33^–^35^ Women have lower levels of alcohol dehydrogenase enzyme (ADH), which results in less efficient alcohol metabolism and elimination from the body.^36^–^38^ Female bodies have lower water and higher fat content than male bodies do, and women reach higher blood alcohol levels (BALs) than men even at the same weight and same amount of alcohol consumed over the same timeframe.^37^,^39^,^40^ Higher BALs may also be partially attributable to interactions between ADH and female sex hormones.^37^,^41^,^42^ The negative health impact of alcohol increases with age; evidence suggests that poorer/slower alcohol metabolism and clearance can contribute to greater impairments in cognition, balance, coordination, and attention for every drink consumed by aging men and women.^9^ Aging women experience greater loss of lean body mass, accelerated increases in body fat, and larger declines in total body water content compared to aging men. Because alcohol distributes primarily into body water, these changes reduce the distribution volume for alcohol in women, amplifying sex differences in alcohol pharmacokinetic at older ages.^37^,^43^ However, research comparing these processes in men and women is lacking.

Menopause is one area of limited research that may be relevant to understanding risk related to AUD and heavy drinking in older women. The menopausal transition is a highly variable life phase in terms of length, symptoms, physical effects, and age of onset.^44^–^46^ A woman is classified as postmenopausal if it has been 12 months since her final menstrual period (i.e., no menses without other medical cause). However, “menopause” can be broken into several sub-phases (i.e., early and late menopausal transition, followed by early and late postmenopause, with the transition marked by the final menstrual period; perimenopause encompasses the early and late menopausal transition phases and the first year of postmenopause). During these phases, endocrine and physical changes occur rapidly and are highly variable.^45^ In particular, the perimenopause phase, which precedes and extends into early postmenopause, can last up to 10 years. During this time, endocrine markers are already highly variable and the physical, mood, and cognitive symptoms traditionally associated with menopause may already begin.^44^,^45^ While the age at which women progress through these phases is widely variable, the median age of final menstrual period is around ages 50 to 52 for white women in industrialized countries.^47^ Therefore, much of the age-related research described in this review coincides with the menopausal transition in older women. One study found that menopause is a period in which many women change their drinking habits.^48^ Unfortunately, none of the studies included in this review examined aspects of the menopausal transition in relation to alcohol use specifically or compare pre- and postmenopausal women, except for some studies that focused on physical consequences of alcohol use (i.e., cancer, bone loss, cardiovascular health). In those studies, findings often did vary between pre- and postmenopausal women, suggesting the potential importance of menopausal status in research on alcohol’s effects and AUD risk.

Ovarian hormones, as well as their precursor hormones and metabolites (e.g., estrogen, pregnenolone, allopregnanolone), also positively impact stress reactivity and cognitive functioning among women, and burgeoning research demonstrates their direct associations with alcohol use among individuals with AUD.^41^,^42^ These hormones circulate at reduced levels after menopause, and therefore, postmenopausal women may not experience their protective or buffering effects. Alternatively, greater alcohol intake is associated with higher levels of endogenous sex hormones, including estrogen, and may impact hormone metabolism and clearance.^49^ Additionally, for some women, menopause is accompanied by significant cognitive, psychological, and physical symptoms,^46^ with up to 60% of women seeking medical care for menopause symptoms.^50^ In combination, these factors may influence alcohol use and alcohol-related consequences in older women. It is important to note, however, that women’s experiences of aging and menopause are highly variable, and individual differences and resiliency factors also must be considered in this research. Nevertheless, most women do naturally decrease their alcohol use with age.^31^ This context is provided here for the reader to consider while reviewing the literature. Ultimately, this work can facilitate future research that more fully accounts for the menopausal transition, a major life phase for many women, in understanding alcohol use and aging in women.

## Objectives

The current scoping review focuses on articles published since 2004 that explicitly included samples of women over age 50 with AUD or heavy drinking. Included studies were required to analyze and present sex difference data, report relevant results for older women, and use rigorous methodology. Findings from wider age- and sex-neutral literatures are integrated in the review, as necessary, to provide context for findings on older women. Methodology was carefully and critically considered in reviewing articles, and suggestions are provided to improve quality and consistency in research on the target population going forward.

## Methods

### Protocol and Registration

An unregistered protocol, available from the corresponding author, was developed to guide this scoping review. The protocol was developed using the Preferred Reporting Items for Systematic Reviews and Meta-Analyses for Scoping Reviews (PRISMA-ScR).^51^

### Eligibility Criteria

English language, human subject, and publication date filters were applied. Initially, 1994 was used as the publication date cutoff, due to documented increases in AUD and alcohol-related problems among women in Europe and the United States starting around the year 2000.^3^,^52^,^53^ As described below in “Critical Appraisal of Individual Sources of Evidence,” however, 2004 was ultimately chosen as the final publication cutoff year.

### Information Sources and Search

Using sentinel articles to harvest and test search terms, the search strategy (Table 2) was developed for PubMed/Medline to retrieve all records using natural language and controlled vocabulary (when applicable) relating to the concepts of alcohol use in older women in articles published since 1994. This search strategy used previously published search strategies for identifying articles on women^54^ and older populations.^55^ In addition to using the sentinel articles to generate this search strategy, several iterations of the strategy were also “tested” by the first author, to confirm that they would yield additional sentinel publications on the topic of alcohol use in older women. The final PubMed/Medline strategy was translated and adapted for the other databases. The following databases were searched for articles published from January 1, 1994, through August 6, 2024: PubMed/Medline (including Pre-Medline and non-Medline), Embase (Elsevier), and PsycInfo (EbscoHost) (Table 2). EndNote v.21 (Clarivate) was used to de-duplicate all records using the method developed by Bramer and colleagues.^56^

### Selection of Sources of Evidence

**Figure 1:**
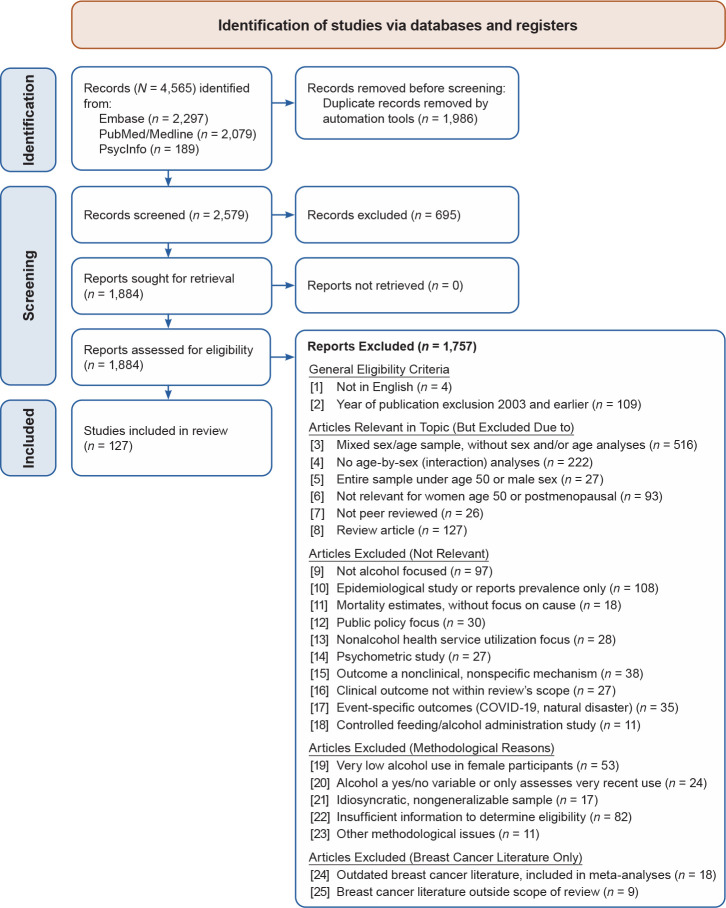
PRISMA flow diagram of the study selection process for correlates, consequences, treatment, and prevention of alcohol misuse in older women. *Source:* PRISMA flow diagram templates are distributed in accordance with the terms of the Creative Commons Attribution (CC BY 4.0) license. Page MJ, Mckenzie JE, Bossuyt PM, et al. The PRISMA 2020 statement: An updated guideline for reporting systematic reviews. *BMJ* 2021;372:n71. doi:10.1136/bmj.n71.

Figure 1 illustrates article inclusion and exclusion throughout each step of the review. Table 3 details all inclusion and exclusion criteria, which are also summarized below. The search strategy identified 2,579 articles. The titles and abstracts of these articles were reviewed for relevance by random combinations of two study team members. After reviewing titles/abstracts, reviewers excluded or included them for the next step of full-text review based on the following inclusion/exclusion criteria. (1) Articles were published in English. (2) Articles were published within search dates. (3) Articles were pertinent to women age 50 and older. This criterion led to exclusion of a large number of studies for several reasons (Table 3). (Note that nine articles were included in the review despite having samples with age ranges that went below age 50. Seven of the nine studies were included because they were longitudinal studies in which participants were over age 50 at follow-up and/or the average sample ages were above the age 50 and older cutoff. The two other studies were unique treatment studies with sample means age 50 and older.) (4) Articles were peer reviewed. (5) Articles were either primary research or meta-analyses. (6) Articles included alcohol-relevant outcomes. (7) Articles provided results beyond the epidemiology/prevalence of alcohol use or overall mortality rates in women compared to men. (8) Findings had clinical implications. (9) Articles did not focus on event-specific outcomes—primarily in relation to the COVID-19 pandemic or natural disasters. (10) Lastly, given the review’s focus on heavy alcohol use and/or AUD among women, any controlled feeding/alcohol administration studies that excluded women with current or past heavy alcohol use/AUD or with a positive family history of AUD were also excluded from this review.

Reviewers agreed on whether to include/exclude 89% of the 2,579 articles and disagreed on 11% of articles based on title and abstract review. The team met weekly to review disagreements and, in the initial few weeks, to calibrate reasons for inclusion/exclusion. Using Covidence software, disagreements were identified and, in all cases, successfully resolved.

For many articles, it was unclear from the abstract whether the criteria outlined above were met, and review of the full text was required. At the title/abstract review stage, 695 articles were excluded, leaving 1,884 articles that required closer review at the full-text stage. A full-text review of each of those articles was completed by one study team member, resulting in exclusion of 1,323 articles that, upon reading the full text, clearly did not meet the inclusion criteria described above. For 561 articles, a critical review was required.

### Critical Appraisal of Individual Sources of Evidence

Upon review of the remaining 561 articles, the cumulative nature of the literature, delayed improvements in the inclusion of female participants, and changes in how sex analyses were conducted led to the decision to focus on the past 20 years of research. Therefore, 108 articles published before 2004 were excluded, leaving 453 articles for possible extraction.

These articles were reviewed by the two senior authors (CGH and EEE), with a focus on critically appraising each article’s methodology. Each article was evaluated based on the following criteria (see Table 3 for summarized criteria):

- Distribution of alcohol use among female participants: When the percentage and number of female participants who consumed alcohol were low, the impact of these low numbers on the study’s results was considered. For example, if 96% of the women in a study did not drink any alcohol, in combined consideration with the overall sample size, the study may have been excluded given very low alcohol use in female participants (Criterion 19).
- Methods used to measure alcohol use or AUD: If participants reported on their alcohol use over just 7 days prior to the study baseline, the study was excluded due to the lack of reliability with such a limited timeframe; if alcohol use was dichotomized as a yes/no variable, to reflect whether a participant consumed any alcohol within any timeframe, the study was excluded (Criterion 20). In some cases, alcohol use was categorized in arbitrary terms (e.g., into low, moderate, and high risk, but not in alignment with NIAAA or other definitions). When possible, reviewers converted these categorizations into NIAAA-defined drinking levels or into standard drinks per day; however, articles for which this was not possible were excluded (Criterion 22 includes these exclusions, as well as other insufficient details, such as number of women in the sample).
- Sampling strategies and sample characteristics: Studies using sampling strategies or samples that significantly limited generalizability of the findings were excluded (Criterion 21).
- Additional methodological issues: An additional set of articles were excluded due to insufficient information to determine eligibility based on the criteria above (Criterion 22) or due to other (i.e., not alcohol-related) methodological issues (Criterion 23), such as limitations of design or statistical power that were identified by both the original authors and the authors of this scoping review.

### Data Charting Process

After critical appraisal, 127 articles were retained for extraction. Data were charted by one of the six authors. Extractions were then reviewed by at least one other author. Extraction was conducted using Covidence software, with the template generated by the study team and led by senior authors (EEE, CGH). One senior author (EEE) met with all other authors to discuss extraction strategies, and five articles were first extracted by all authors to calibrate on level of detail and content included. After calibration, authors independently charted data but brought questions regarding individual articles to weekly meetings for discussion.

### Synthesis of Results

The template of the extraction tables (Appendices 1 to 4) was designed to facilitate synthesis of results, by topic. Given the broad and comprehensive nature of this scoping review, each author focused on specific topic section(s), but all sections were edited by the senior authors. Each author reviewed extraction summaries and section write-ups to further synthesize findings.

## Results

### Biological Correlates of Alcohol Misuse in Older Women

The studies retrieved for this scoping review on biological, psychological, and social correlates of alcohol use and misuse in older women; their main characteristics; and their main findings are summarized in Appendix 1.

Seven studies examined the correlational relationship between alcohol consumption and general health among older women.^57^–^63^ These articles did not examine health outcomes of alcohol use, but rather examined the correlational presence of health issues, chronic disease, and health-related mechanisms among women who drank alcohol at varying quantities. In terms of general health, five of the seven correlational studies found that women who drank alcohol self-reported having better general health than women who did not drink.^57^–^61^ However, these articles either did not account for women who quit drinking,^57^,^58^ and/or found ceiling effects whereby drinking more than two drinks per day^60^ or experiencing alcohol-related problems (per the Alcohol Use Disorder Identification Test-Concise [AUDIT-C])^61^ mitigated any findings of better self-reported health among participants. One study found that women who consumed one or more drinks per day or seven or more drinks per week (versus less than one drink per day and less than seven drinks per week) self-reported better health, but only compared to those who had formerly been drinking and not compared to women who never drank.^59^ That study additionally found that the heavier drinking group reported fewer heart or cholesterol problems, but were also more likely to report unhealthy behaviors such as cigarette use or living a sedentary lifestyle.^59^

Three correlational articles on sleep and pain in relation to alcohol use among older women were identified.^61^–^63^ Only one study assessed the associations of pain and alcohol use in older women.^62^ Women ages 55 to 65 who reported one or more alcohol-related problems on the Drinking Problems Index (DPI) were more likely to use alcohol for pain management, compared to women who denied any alcohol-related problems; more frequent drinking to deal with pain at baseline was then associated with more alcohol problems at 3-year follow-up.^62^ Use of alcohol as a sleep aid, shorter latency to sleep,^63^ and use of sleeping pills,^61^ as well as regular use of more than one over-the-counter medication,^63^ were all associated with alcohol-related problems on the DPI or AUDIT among women age 60 and older.

#### Summary

These correlational studies on self-reported health, medical conditions, and alcohol use suggest a potential “sick quitter” effect, wherein alcohol abstinence in older age may be due to experiencing alcohol-related health problems earlier in life.^64^ This effect likely contributes to better self-reported health among women who drink, particularly for those who drink alcohol with relatively low frequency and intensity. These studies are correlational and do not indicate any health benefit of alcohol use among women. Additionally, given that these studies were conducted among general samples of women age 50 and older, findings may indicate that developing chronic health conditions—whether they are alcohol related or not—or perceiving one’s health as poor may motivate women to quit drinking with advancing age. In turn, this contributes to the “sick quitter” effect in research with older women, which needs to be accounted for in research methods and designs with this population. Future studies that focus on women with AUD or at risk for AUD may also find different results, such that health may be differentially associated with decisions about alcohol use.

Older adults are more likely to experience worse sleep and acute and chronic pain than younger adults,^65^ regardless of alcohol use.^66^–^70^ Additionally, older women may be particularly susceptible to insomnia and pain,^71^ both of which are common antecedents for alcohol use and exacerbate AUD sequalae.^5^,^68^,^71^ Additional research on these topics among older women is critical and may identify opportunities for screening, prevention, and treatment efforts.

### Psychological Correlates of Alcohol Misuse in Older Women

#### Distress, mood, and stress

Outside of co-occurring psychiatric conditions, six articles explored the role of self-perceived stress or psychological distress in relation to alcohol use, binge drinking, or AUD.^72^–^77^ In terms of general psychological distress, women who consumed three or more drinks on one occasion in the past month reported greater distress compared to those who did not drink at all, which was not found among men.^73^ For women consuming up to two drinks per day, however, there was no association of drinks per day with distress.^74^ Alternatively, when looking at AUD in a sample age 60 and older, higher perceived stress was associated with higher risk for AUD among men but not women.^75^ These studies differed in terms of alcohol-related outcome (drinking versus AUD) and stress measure (perceived stress versus psychological distress) assessed, and were conducted in different age groups (age 50 and older versus age 60 and older). None of the studies accounted for former or lifetime alcohol use patterns.

Three studies found positive associations of stressful life events, including adverse childhood experiences, and risk for AUD in both men and women age 50 and older.^75^,^76^,^78^ All studies were among general population samples and therefore included both male and female participants as well as individuals who no longer consumed alcohol for unspecified reasons. A fourth study only analyzed data among people age 50 and older who currently consumed alcohol; it found that living in adverse neighborhood conditions (i.e., neighborhoods characterized by relatively higher numbers of 911 calls, violent crimes, families living below poverty level) was associated with binge drinking in women but not men.^77^

One study among men and women age 55 and older conducted latent class analyses among those engaging in “heavy drinking” (which, given their standard drink definition, translated to consuming six or more U.S. standard drinks per week for women and 12 or more U.S. standard drinks per week for men).^72^ The study found that women ages 55 to 64 who drank heavily were more likely than their male counterparts to report distress due to pain, sleep, and tiredness; moreover, women ages 65 to 74 who drank heavily were more likely than male counterparts to report distress and impairment related to pain and physical health.

##### Summary

Age-neutral research has found a strong relationship between stress, heavy drinking, and/or AUD among women, with higher levels of psychological distress increasing AUD risk to a greater extent for women compared to men.^17^,^79^ Sex differences also exist in the relationship between trauma exposure and AUD risk among general adult populations, with most research showing a stronger association of traumatic experiences and risk of AUD (and other substance use disorders) among women compared to men.^17^,^80^ The preliminary research among older women reviewed here has yielded similar findings, particularly when focused on older women who are not abstinent from alcohol, and less so for studies that use national survey data. This body of literature is very limited, however, and continued research will help to further elucidate these relationships.

#### Psychiatric comorbidities

##### Cross-sectional and correlational studies

Five studies used cross-sectional data to examine psychiatric comorbidities in relation to alcohol use or AUD among older women.^78^,^81^–^84^ In a latent class analysis of men and women age 50 and older who self-reported being troubled by an alcohol-related problem on the Addiction Severity Index in the past month, women were more likely than men to belong to a class of alcohol use characterized by co-occurrence of depression or anxiety and emotional and physical abuse trauma.^78^ Two studies found that, among women age 65 and older, higher scores on the AUDIT were associated with higher rates of depression and self-reported likelihood of drinking to cope with depression.^81^,^83^ Among people who were currently drinking, women age 50 and older who binge drank less than monthly in the past year reported higher rates of panic disorder and post-traumatic stress disorder (PTSD) than did women who did not binge drink or who binge drank monthly.^84^ Monthly binge drinking also was associated with higher likelihood of AUD.^84^ A separate study found that, after controlling for all other lifetime diagnoses according to the *Diagnostic and Statistical Manual of Mental Disorders, 4th Edition* (DSM-IV), women age 65 and older with a lifetime mood disorder had an increased risk for current and lifetime DSM-IV alcohol abuse and/or dependence.^82^ In another study of patients age 60 and older who sought treatment for depression at an outpatient clinic, 27% reported having consumed five or more drinks on one occasion in the past year (compared to 32% of male counterparts in the clinic), while 13% used cannabis (14% in men), 18% used sedatives (other than as prescribed; 21% in men), and 16% used tobacco in the prior year (14% in men).^85^

##### Longitudinal studies

Four studies further examined the association between alcohol use and depression and anxiety using longitudinal data, with each examining different aspects of alcohol use.^86^–^89^ A pooled analysis of longitudinal studies found that women (in this case, age 45 and older) who were abstinent or consumed 1.5 to 3.0 drinks per day had higher likelihood of depression compared to women who drank less than 1.5 drinks per day.^89^ Moreover, women who were abstinent across three study timepoints had increased odds of depression compared to those who drank less than three drinks per day. In another study, women (and men) ages 57 to 65 who consumed seven drinks or less per week (i.e., were within the *Dietary Guidelines for Americans, 2020–2025*) were at lower risk of developing depression symptoms over an average of 8 years compared to those who never drank.^86^ Risk of depression did not differ between those who never drank, those who had been drinking but had quit, and those who drank heavily (i.e., more than seven drinks/ week). The authors highlighted several limitations, including low rates of heavy drinking and survey nonresponse at follow-up. Another 10-year study of pooled survey data from older men and women (age 50 and older) found that women who drank alcohol at any level had higher incidence of depression than any men.^88^ Depression risk was heightened for women who drank any alcohol even when compared to men who drank moderately (defined as weekly drinking but drinking three drinks or less per day and no binge drinking). In a third study looking at individuals with alcohol misuse (defined as having a score of at least 2 on CAGE screener, see Table 1), older women but not older men had higher risk of depression and anxiety compared to their counterparts who did not drink.^87^ When alcohol use was categorized according to drinking intensity, there were no differences between people who did not drink (only assessed in the past 6 months) and people with different drinking levels (less than four drinks per day, four or more drinks per day, or 10 drinks per week).^87^ Thus, symptoms consistent with AUD on the CAGE were associated with depression and anxiety in older women but intensity of drinking was not. This latter study was the only one of the three longitudinal studies that did not exclude participants with depression or anxiety at baseline and problem drinking (per the CAGE score of at least 2) also increased the likelihood of persistent depression over 2 years in women.^87^,^90^

###### Summary

Age-neutral research has consistently found that women with AUD have higher rates of co-occurring mood and anxiety disorders compared to men with AUD.^90^ Although the summary above reflects a general paucity of research on the psychiatric comorbidities in older women with AUD, findings suggest that these patterns may persist into older age. The cross-sectional studies had a number of limitations, including not accounting for lifetime alcohol use; however, some studies did focus on people who currently consumed alcohol and/or women with heavy drinking. Cross-sectional studies also generally failed to include direct sex comparisons and instead analyzed data for men and women separately. Two longitudinal studies^87^,^88^ directly compared older men and women and found an association between alcohol use, alcohol misuse, and depression among women but not men. While limited, findings from the longitudinal research suggest that older women with AUD or who experience problems related to their alcohol use may be at heightened risk of co-occurring depression and/or anxiety. Experiencing alcohol-related problems may be more strongly associated with depression/anxiety risk than drinking intensity or frequency; however, heavy drinking, as defined by NIAAA, seems to also carry risk for older women.

#### Clinical presentation and course

Eight articles discussed the clinical course of alcohol misuse in older women.^78^,^91^–^97^ Five articles examined the progression of alcohol use for older adults over time.^92^–^94^,^96^,^97^ These studies followed a general population of adults over 10 to 20 years. Results indicated that alcohol use decreased with age for men and women,^92^–^94^,^96^,^97^ particularly in intensity, with some studies showing an increase in frequency.^92^,^93^,^97^ Women age 50 and older seemed to decrease their alcohol use more slowly than men did^91^ or have more stable drinking patterns over time,^93^ which may reflect stably low drinking patterns. Women were also more likely to transition to abstinence in older age than men did.^93^,^96^ One study suggested that several AUD-related symptoms at baseline (e.g., drinking to cope, having a heavy-drinking social network) may increase likelihood of women quitting between ages 55 to 75;^97^ however, analyses did not account for the loss of these participants due to health issues or mortality.

The other three studies focused on individuals with AUD and alcohol-related problems. Older women, compared to older men, were more likely to have a late onset of AUD (i.e., onset of regular and heavy drinking at ages 40 and 45, respectively, whereas in men, an onset in their early 20s was more typical).^78^,^95^ Treatment-seeking women age 60 and older with AUD also endorsed more AUD symptoms than their male counterparts, including irresistible cravings and loss of control over drinking.^91^

##### Summary

AUD among women of all ages has historically been characterized by a “telescoping effect,” such that women tend to initiate alcohol use at a later age than men but escalate more quickly to AUD, possibly as a result of compounding biopsychosocial risk factors.^98^ While still a burgeoning area for research, the studies among clinical samples described above^78^,^91^,^95^ seem to replicate those findings in samples of women age 50 or older. Findings such as these, which support late onset and high acuity of alcohol misuse in older age, begin to align with previous findings. However, the current research focused on clinical samples is insufficient as the studies do not adequately speak to the progression of alcohol use across time, including age of onset and rate of increase. Additionally, these studies are prone to survivor bias, particularly given the high morbidity and mortality associated with severe AUD among young and middle-aged women.

#### Co-occurring tobacco use

Five articles addressed co-occurring tobacco use as a correlate of alcohol misuse in older women.^58^,^82^,^96^,^99^,^100^ Higher levels of tobacco use were consistently associated with more frequent and intense alcohol use. Specifically, tobacco use was associated with binge drinking (defined as four to five drinks on a single occasion, depending on the study),^58^,^96^,^99^ increasing frequency of drinking in a longitudinal study, and co-occurring heavy alcohol use.^96^,^100^ Current and lifetime tobacco use disorder was three times more common among both men and women over age 65 with AUD than those without AUD.^82^ When comparing older women with older men, these associations were generally found in both sexes.

##### Summary

Findings on co-occurring tobacco use and alcohol use and misuse were predominately derived from large epidemiological studies. Given extensive research on exacerbated harms of alcohol use in combination with cigarette, tobacco, and/or nicotine use, this is an area in need of continued research and intervention work for older women (and men). As discussed below in the “Consequences” section, the combined use of these substances significantly elevates risk for several diseases and conditions in older women, further warranting additional research and intervention.

### Social Correlates of Alcohol Misuse in Older Women

Seven studies assessed associations between marital status and alcohol use in older women.^58^,^61^,^94^,^96^,^99^,^101^,^102^ Of these, four studies found such associations,^61^,^94^,^101^,^102^ while three studies did not.^58^,^96^,^99^ Studies that found an association showed that women living with a partner were more likely than women who were divorced or widowed to have AUDIT-C scores of 3 or higher, indicating potential alcohol misuse based on quantity and frequency of drinking and binge drinking.^61^,^101^ However, women who lived with a partner were also less likely than those not living with a partner to report alcohol-related problems on the full AUDIT (problems which are largely consistent with DSM-5 AUD symptoms),^61^ indicating that associations may differ based on the outcome of interest (e.g., drinking patterns versus AUD symptoms). Older women who had no partner or who had separated or lost their partner across a 13-year period had a sharper decline in drinking frequency and intensity, compared to women with a romantic partner throughout that time.^94^ Another study found that marriage, including remarriage, increased older women’s heavy drinking relative to those who were never or previously married.^102^ Moreover, women who divorced at a relatively older age more quickly decreased their heavy drinking after divorce, compared to women who divorced in younger years, while stably married women drank most heavily.^102^ Thus, divorced women at age 60 were more likely to drink heavily than stably married women at age 60, but after age 60, heavy drinking declined faster with age for the divorced than for stably married women. These results suggest that studies of longitudinal changes in drinking related to relationship status may provide more nuanced results than cross-sectional studies. Moreover, the existing studies did not differentiate the reasons why participants had lost their partners (e.g., through divorce, separation, death), which may have important implications for changes in alcohol use in older women. One study found that women age 60 and older in AUD treatment were more likely than their male counterparts to be widowed and living alone, and to have higher rates of retirement.^95^

The effects of social networks on drinking behavior in older adults may depend on relationship quality and who is in their social network, especially for women; however, research assessing these associations was limited.^103^,^104^ In one study, increases in social integration and positive quality of social interactions were strongly associated with fewer binge drinking days, whereas negative social support was associated with more binge drinking days among women over age 50, but not among men.^103^ Alternatively, among lesbian and bisexual women age 50 and older, those with greater social support were more likely to engage in heavy drinking than to be abstinent.^104^

Education was a commonly examined correlate and higher levels of education were associated with a greater likelihood of heavy and binge drinking among older women in some studies.^58^,^61^,^101^,^105^ Other studies found that more years of education were associated with fewer binge drinking days; however, “binge drinking” was inconsistently defined as either four or more or five or more drinks per occasion.^99^,^103^ Four studies showed that older women with more education consumed more alcohol than those with less education, however the association between education and alcohol use may not be a linear one.^61^,^94^,^96^,^106^ One study found that women age 70 and older were more likely to have stopped drinking than to currently engage in heavy drinking if they were educated beyond secondary school.^100^ In summary, more education generally was correlated with more alcohol use among older women, and most of these studies did not find any association of education with alcohol use among men. Importantly, only some of these studies controlled for covariates as described in the summary below regarding the role of socioeconomic status.

Only one study examined the impact of retirement on drinking frequency/intensity and reported no association among women age 50 and older.^77^ Of course, retirement does not broadly increase likelihood of drinking for all older adults; however there was no research that examined the associations of retirement with drinking for women who may already have been at risk for alcohol misuse in older age. Findings on the association of income and alcohol use in older women were mixed. Two studies suggested that higher income was associated with more frequent, but not more heavy, alcohol use and that women with the lowest household incomes may drink equally or less frequently than women with higher incomes but may engage in more binge drinking.^94^,^99^ A sample of lesbian and bisexual women age 50 and older with incomes of more than 200% of the federal poverty level were more likely to engage in heavy drinking than to be abstinent.^104^ One study found that the association of income with past-month binge drinking in older women was no longer significant after controlling for demographics and general health.^58^

#### Summary

The research reviewed here on the associations between marital status, relationships, and social support with drinking among older women is consistent with age-neutral research on the topic,^107^ indicating the relevance of social networks to women’s alcohol use and AUD risk. However, the directionality of effects in older women is less clear. The associations of socioeconomic status (SES; e.g., education, income) with alcohol use and alcohol-related consequences are nuanced. For both men and women over age 50, the association between physical health and alcohol use follows the same patterns as for SES, suggesting confounding covariance between SES, alcohol use, and health.^108^ This is consistent with the “alcohol harm paradox,” which finds that people at higher SES drink more alcohol while experiencing less severe consequences from their drinking than people at lower SES.^109^ Thus, research on alcohol use in relation to education, income, and retirement is limited, potentially confounded with other variables (including SES), and thus warrants further investigation.

### Physical Consequences of Alcohol Use and Misuse in Older Women

The studies retrieved for this scoping review that summarize the physical, cognitive, and neuropsychological consequences of alcohol use and misuse in older women; their main characteristics; and their main findings are summarized in Appendix 2.

#### Alcohol and cancer

A 2025 U.S. Surgeon General’s report highlighted alcohol use as a leading preventable cause for six types of cancer.^110^ There is a linear association of alcohol use with cancer risk, with even small amounts (e.g., less than one drink per day) being associated with increased risk of certain types of cancer (e.g., breast cancer; see below for details).^110^ Accordingly, in 2015, the fourth edition of the European Code Against Cancer, part of the World Health Organization (WHO) International Agency for Research on Cancer, revised its recommendation on drinking from, “Moderate your consumption to two drinks per day if you are a man or one drink per day if you are a woman”^111^ to “Not drinking is better for cancer prevention.”^112^ In a 2026 fifth edition, this was further edited to “Avoid alcoholic drinks.”^113^ Suggestions for similar revisions in messaging have been made in the United States.^114^ The Surgeon General’s report does not mention age effects on alcohol-related cancer risk, nor does it mention changes in cancer risk after menopause, although much of the research has specifically recruited postmenopausal women (and, in some cases, compares pre- and postmenopausal participants, as described below).

The age-neutral literature on alcohol-related cancers is large; although the inclusion/exclusion criteria of this scoping review significantly narrowed the number of articles, many articles related to breast cancer remained. Thus, full coverage of that literature is beyond the limits of this review (see Freudenheim 2020^115^ for a comprehensive review of age-neutral research on this topic). The following sections focus on meta-analyses that attend to age or menopausal status as a moderator of outcome and on primary research among older women that was not otherwise reviewed in meta-analyses (see additional exclusion criteria in Figure 1).

##### Alcohol use and breast cancer risk

The most consistent finding across the meta-analyses and studies included in this review was that more frequent and intense alcohol consumption was linearly associated with increasing risk of developing postmenopausal breast cancer.^116^–^126^ Whereas most studies did not account for changes in alcohol use over time in relation to risk, one study found increased breast cancer risk among women who had previously used alcohol compared with those who never drank.^125^ In terms of dose effects, a meta-analysis^123^ found that postmenopausal breast cancer risk increased by 11% for every 0.7 drinks per day of total alcohol consumption and 23% for each additional 1.4 drinks. These findings align with a separate report from the World Cancer Research Fund (not extracted in this review), indicating an 8% increase in relative risk for a 0.7 drink/day increment of alcohol consumption among postmenopausal women.^127^

Several articles examined the association of alcohol use with increased risk of specific breast cancer subtypes, including one meta-analysis.^128^ Although other breast cancer subtypes exist, the articles extracted in the current review were focused on breast cancer subtypes based on hormone receptor status (i.e., tumors that are estrogen receptor-positive or -negative [ER+/−] and progesterone receptor-positive or −negative [PR+/−]) and on histological subtype (i.e., present in lobules and/or ducts). Some studies found that the increased cancer risk seen with higher alcohol consumption was strongest, or restricted to, breast cancers with ER+ tumors,^117^ ER+/PR+ tumors,^121^,^122^ or ER+/PR− tumors.^122^ Findings of increased risk for hormone-receptor positive tumors with more alcohol use applied to women of all ages,^128^ but the association appeared stronger among postmenopausal women compared to premenopausal women.^96^ Studies examining histological subtypes of breast cancer found evidence of increased alcohol-related risk for all types in postmenopausal women, including ductal,^121^ lobular,^121^,^122^ and both or mixed tumors.^121^,^125^ However, findings were mixed, with one study showing higher risk only for lobular tumors.^122^

Several studies examined putative mediators of alcohol’s effects on breast cancer development, including effects on elevated estrogen levels; disrupted folate metabolism and its impact on one-carbon metabolism, which is critical for epigenetic regulation; levels of the carcinogenic alcohol metabolite acetaldehyde; dietary deficiencies; and cancer-promoting inflammatory cytokines (e.g., C-reactive proteins).^117^–^120^,^124^,^126^,^129^,^130^ The interactions of alcohol with certain genotypes to directly impact carcinogenesis or alcohol metabolism were also examined.

Among postmenopausal women, genetic polymorphisms that are critical to one-carbon metabolism were associated with increased breast cancer risk for women consuming two or more drinks per day, compared to nondrinking women with the same genetic constitution.^129^ The *ADH1B* genotype (which encodes the alcohol-metabolizing alcohol dehydrogenase 1B) interacted with any alcohol use to increase cancer risk,^119^ although these results were mixed^120^ and potentially based on the genetic single-nucleotide polymorphism tested. Another study found that genes related to C-reactive protein interacted with high alcohol intake and other lifestyle factors to increase risk.^130^ Lastly, the increased risk of breast cancer due to alcohol use among postmenopausal women was found to be stronger among those with low dietary folate intake.^118^

There is also evidence of increased risk among women who use hormone therapy and consume alcohol at heavy drinking levels, compared with alcohol-abstinent women who do not use hormone therapy.^124^,^126^ This increased risk of breast cancer in relation to alcohol use and hormone therapy is thought to be likely due to elevated estrogen among women who drink alcohol or drink heavily. A 2023 meta-analysis examined endogenous hormone levels in relation to alcohol use, but did not look at breast cancer outcomes.^49^ The meta-analysis found that higher alcohol intake was associated with higher levels of estradiol (and several other sex hormones) but lower levels of sex-hormone binding globulin in postmenopausal, but not premenopausal women, promoting higher levels of circulating hormones in postmenopausal women. Further examination in this study of the ADH1B gene found that alcohol may influence hormone metabolism and clearance, with potential shared genetic underpinnings of alcohol and hormone metabolism, which could create interactive risk for negative health outcomes, including cancer.^49^

One study also examined breast density, another putative mediator of alcohol’s effects on breast cancer risk. The analysis found that absolute breast density volume mediated 25% of the effects of alcohol intake on breast cancer risk among postmenopausal but not premenopausal women.^117^ Overall, these studies suggest several potential mechanisms that account for the alcohol–breast cancer association.

##### Alcohol use and risk for other cancers

In addition to breast cancer, the Surgeon General’s report noted alcohol-attributable risk for colorectal, liver, and four types of aerodigestive cancer: esophagus, mouth (oral cavity), throat (pharynx), and voice box (larynx).^110^ This scoping review’s full-text screening identified five articles that examined alcohol-attributable risk for aerodigestive, colorectal, endometrial, or skin cancer among older women.^131^–^135^

One study—the U.K. Million Women Study that included participants age 50 and older—found strong associations of alcohol use with aerodigestive disorders, with linear increases in the risk of esophageal squamous cell carcinoma (44% increase), oral cavity and pharynx cancer (36%), and larynx cancer (35%) per every 0.6 drinks of daily alcohol consumed; risks were increased more for postmenopausal women who also smoked.^131^ Women who consumed eight or more U.S. standard drinks per week and smoked 10 or more cigarettes per day were 9.7 times more likely to develop any aerodigestive cancer compared to those who never smoked and drank one to two drinks per week, with linear increases in risk for women who drank and smoked at levels in between. There was also increased risk of breast, colorectal, pancreatic, and lung cancers among women who drank in this sample. At the same time, for every 0.6 drinks per day increase in alcohol intake, there was a *decrease* in risk of other cancers, including thyroid cancer, renal cell carcinoma, non-Hodgkin’s lymphoma, and multiple myeloma. However, the authors noted that only 8% of their sample (corresponding to about 63,600 women) fell into the highest category of alcohol use (i.e., the equivalent of approximately 8.5 U.S. standard drinks per week), which is notable and may have implications for findings.

A prospective cohort study on colorectal cancer found no association with alcohol use.^132^ The parent study cited low levels of alcohol use among its female participants, and its analyses used women who did not drink as a reference group, without distinguishing between those who had been drinking previously and those who never drank. Another study found associations between baseline alcohol consumption and risk for melanoma and nonmelanoma skin cancer.^133^ Women with heavy drinking at baseline had 64% higher risk of melanoma and 23% greater risk of non-melanoma skin cancer compared to those who did not drink during the 10-year follow-up. Lifetime alcohol consumption (i.e., more drinking years) was also associated with elevated risk for both cancer types when compared to women who never drank.

Two prospective cohort studies on endometrial cancer risk yielded conflicting results. In one study, postmenopausal women who consumed 1.7 drinks per day had twice the risk of endometrial cancer over 8 years follow-up compared with women who did not drink.^134^ The second study, however, did not find any association between alcohol intake and endometrial cancer risk using the same alcohol metrics; however, this sample included 10% premenopausal women.^135^ Both studies compared groups based on drinks per day, and neither distinguished women who never drank from those who had previously consumed alcohol.

###### Summary

Age-neutral research strongly indicates a linear association of alcohol use with breast cancer risk,^115^ which is also supported by this literature among older and postmenopausal women specifically. The research covered here on alcohol and cancers other than breast cancer is extremely limited, and results should be interpreted cautiously. Most of the latter studies compared categories of alcohol consumption to women who did not drink, without distinguishing whether they had quit drinking or had never drank. Additionally, many of these studies did not have primary aims focused on alcohol use in relation to cancer, and therefore did not conduct targeted recruitment efforts, resulting in relatively low numbers of women who drank heavily in some of the samples; this was found in breast cancer studies as well. Many of the studies also cited high numbers of White participants, with underrepresentation of individuals from other racial or ethnic backgrounds. As described below in the “Prevention” section, one study conducted qualitative research with women ages 40 to 65 to develop an intervention focused on the association between alcohol consumption and breast cancer risk.^136^ Based on those interviews, the study’s authors suggested that prevention interventions should tailor information to women’s experiences, take into account and address the perceived social benefits of alcohol use (e.g., acknowledge that some women view drinking as routine or integral to their social interactions), teach healthy coping strategies, and avoid judgmental or patronizing language.^136^ Including simple statistics regarding alcohol-related risks and providing healthy alternatives were also highlighted as potentially useful avenues. Given the evidence for alcohol-related increases in breast cancer risk especially (and likely, other cancers) in older women, more such studies may provide additional evidence to inform prevention and intervention efforts.

#### Other disease outcomes

##### Cardiovascular disease

The literature identified for this scoping review on alcohol consumption and cardiovascular disease risk among older women is ambiguous, due to inconsistent methodological rigor. Five articles on cardiovascular consequences of alcohol use among older women met inclusion criteria; many others were excluded due to methodological issues (beyond those described here).^137^–^141^ Two articles on alcohol consumption and stroke risk indicated mixed results among older women. One study found higher stroke risk among women who drank more than 1.7 drinks per day compared to those who consumed less than 0.4 drinks per day; however, analyses did not clearly account for lifetime drinking patterns or for individuals who may have quit drinking before the study.^137^ The other study, which did account for these factors, showed no difference in stroke risk based on drinking habits, but former drinkers had increased stroke risk compared to current drinkers.^138^ Both studies had relatively low numbers of female participants who drank alcohol.

Analyses of other indicators of cardiovascular health also yielded mixed results. Women age 45 and older who consumed two or more drinks per day seemed to have an increased risk of atrial fibrillation compared to those who did not drink (without differentiating between those who never drank and those who had quit drinking).^139^ Among women ages 65 to 88 with hypertension, those who consumed one to seven drinks per week or more than eight drinks per week had no increased risk of heart failure during 11 years of follow-up compared to those who never drank.^140^ One article focused on the association of alcohol with mechanisms of cardiac disease (e.g., carotid plaques, arterial thickness and diameter) found associations of drinking patterns with certain measures, but not others.^141^ Aside from the overall scarcity of papers, each of these studies had important limitations. One common limitation was that study samples generally comprised relatively healthy female participants in terms of alcohol use, with few who consumed alcohol at all or were heavy drinkers. Given the different outcomes assessed and the limitations, it would be difficult to draw any consistent conclusions regarding alcohol use and cardiovascular health among older women.

##### Liver disease

The relationship between alcohol use and liver-related medical events (e.g., a medical encounter for liver disease) was observed among postmenopausal women ages 50 to 74 in one prospective cohort study.^142^ The lowest risk of a liver-related event was observed among women who drank up to approximately 8.5 drinks per week, compared to women who abstained (with no differentiation for those who had quit drinking) or who drank more than approximately 8.5 drinks per week. A second study found no linear relationship between alcohol use and elevated alanine and aspartate levels, both of which are serum markers of liver disease.^143^ Notably, and in contrast to the first study’s findings,^142^ this second study found that alcohol use interacted with body mass index, suggesting a synergistic effect of obesity and alcohol on liver function among women (and men). Both studies used a “nondrinker” group as the referent category in analyses, without distinguishing between those who had abstained throughout their lifetime, those who had quit drinking, and even those who consumed alcohol but did not drink at least weekly, posing a significant limitation with this research.

##### Musculoskeletal conditions

Four articles on orthopedic consequences of alcohol use included samples of postmenopausal women with mean ages over 55.^144^–^147^ One study focused on muscle health and three articles examined bone health, each using different outcome measures. None of the studies accounted for potential former alcohol use or lifetime drinking habits. The study on sarcopenia (i.e., muscle loss) found that the prevalence rates of sarcopenia were 8%, 11%, and 23% among postmenopausal women with AUDIT-K (Korean translation, with scores consistent with original AUDIT) scores in the ranges of “low-risk,” “hazardous/harmful,” and “likely alcohol dependence” levels, respectively.^144^

With respect to bone health, women who did not drink alcohol or who drank more than twice per week had lower bone mass densities and 1.7-times greater risk of osteoporosis compared to women drinking monthly but less than twice per week.^145^ In another study, consuming about six drinks per week or more was associated with both detrimental and beneficial effects on measures of bone health among women.^146^ However, because few women in the study drank at that level and no distinction was made for former drinking habits, these results should be interpreted with caution. One experimental study asked women who regularly consumed about 1.4 drinks daily to abstain from alcohol for 14 days and then to resume drinking at their typical levels in an effort to examine changes in markers of new bone formation and resorption.^147^ Abstinence contributed to increased markers of bone turnover (i.e., greater bone resorption compared to bone formation), and reinitiation of alcohol use led to reduced bone turnover. The authors posited that this inhibitory effect of alcohol on bone turnover may explain why alcohol use among postmenopausal women may promote increased bone density. Collectively, the findings suggest a potentially complex relationship among alcohol use, bone health, and sex, but methodological limitations and limited numbers of studies suggest a need for additional work.

##### Other health outcomes

Three articles examined alcohol use and mortality.^148^–^150^ There was a nonlinear association between alcohol consumption of more than two drinks per day and increased risk of all-cause mortality among older women.^148^ Those who increased their number of weekly drinks after age 50 had higher mortality risk compared to women who had stable drinking patterns after age 50; however, the study did not account for changes in drinking earlier in life.^149^ Among women ages 50 to 79, the rate of alcohol-related mortality was higher (controlling for age) for women living alone, compared to married or cohabitating women;^150^ however, the directionality of these associations is unclear, and isolation and alcohol use may have interactive effects on mortality risk.

Between 2011 and 2019, rates of emergency room visits for alcohol-related falls increased in women age 65 and older. Increases occurred in all age subgroups (65 to 69, 70 to 74, 75 to 79) except those age 80 and older; the highest rate of increase was among women age 70 to 74, with an annual percent increase of 15% (i.e., in this group, rates increased at an average rate of 15% every year between 2011 and 2019).^151^ The article did not examine reasons for increasing alcohol-related falls among older women, but hypothesized that they may be attributable to increased rates of alcohol use, physiologic factors (e.g., less lean muscle mass) that lead to higher BALs in older women, and more common use of medications such as antidepressants and benzodiazepines, which—particularly when combined with alcohol use— exponentially increases fall risk.^151^ Two articles found increased risk of accidents and related mortality for women age 55 and older who drink compared to those who do not drink.^152^,^153^ Thus, older women with a diagnosis of alcohol abuse according to the International Classification of Diseases^28^, 9th Edition in Medicare claimant data had an increased risk of a fall-related injury compared to those without this diagnosis.^152^ Also, women age 55 and older who drank 12 or more alcoholic beverages in the past year (versus those who did not drink any alcoholic beverages) were more likely to experience fatal falls and motor vehicle accidents, and die by suicide, with the increased risk of suicide being a larger effect among women than men.^153^

Among women age 70 and older, those consuming approximately 0.3 to 3.0 drinks per day had higher odds of “successful aging” (i.e., being free of 11 major chronic diseases and of physical, cognitive, or mental health impairments or limitations) over 16 years of follow-up than did abstinent women.^154^ Spreading alcohol consumption throughout the week was associated with higher odds of successful aging, whereas drinking on just 1 or 2 days was not.^154^ A second study found that higher drinking frequency (i.e., drinking weekly versus monthly) and drinking intensity (i.e., more versus less than 1.4 drinks per day) led to faster health decline over 10 years.^155^ However, neither study accounted for history of alcohol use, limiting the conclusions that could be drawn regarding alcohol use and physical decline in older age. As described throughout this scoping review, particularly in this section on “Alcohol and Other Disease Outcomes,” the limited representation of women with the highest levels of alcohol consumption contributes to an incomplete understanding of the relationship between alcohol use and physical functioning. The findings from these two longitudinal studies correspond with findings from cross-sectional data, described above in the section “Biological Correlates of Alcohol Misuse in Older Women,” indicating that poor health may be a motivator for quitting drinking as women age in the general population. This issue remains largely unstudied among women who exhibit heavy drinking or are otherwise at high risk for AUD and alcohol-related disease.

###### Summary

The literature included in this review that was focused on physical consequences of alcohol use in older women was very limited and precludes definitive conclusions or summaries of results in most areas examined. Existing research is limited by an overreliance on secondary analysis of large datasets, and many studies do not account for participants’ histories of alcohol use; changes in alcohol use over time; or important covariates related to social, emotional, and overall health. Future research that builds on age-neutral, female-specific research in these areas (e.g., Piano et al., 2020^156^) may help clarify risk associated with heavy drinking and AUD for disease outcomes based on changes in women’s alcohol use after age 50. Such research could inform prevention efforts for the growing population of older women with alcohol misuse.

### Cognitive and Neuropsychological Consequences

Three studies examined the cross-sectional relationship between alcohol use and cognition.^157^–^159^ One study found that, among women ages 65 to 85, consuming one to two drinks per day was associated with better scores on self-report and objective assessments of cognitive performance (including measures of global cognition, memory, and executive functioning) and lower risk of Alzheimer’s disease, compared to abstinence or drinking three or more drinks per day.^157^ Among women age 50 and older with hypertension, a higher AUDIT-C score was associated with higher levels of subjective distractibility.^158^ Across studies, authors have highlighted the need for additional high-quality research to better understand the unique and combined effects of metabolic, genetic, and brain-related factors on the relationship between alcohol and cognition. A third study on the association between alcohol use and episodic and semantic memory stores yielded null findings.^159^ This suggests that ambiguous or contrary results, such as those reported here, may be at least partially attributable to confounding factors such as demographic variables that are not often controlled for (e.g., medical conditions or mood disorders). Alternatively, different associations likely exist between cognitive outcomes— whether neuropsychological measures or risk for dementia and related diseases—in relation to alcohol use versus alcohol consequences. Additionally, none of these studies, all with older adult samples, accounted for lifetime alcohol use or former drinking in their study design or analyses.

Nine studies used longitudinal datasets to examine alcohol use and cognitive outcomes among older adults, with a range of 2 to 14 years of follow-up data.^160^–^168^ None of these studies assessed or accounted for lifetime history of alcohol use. As in the cross-sectional research, a diagnosis of AUD was consistently associated with increased risk of dementia and/or Alzheimer’s disease among older women.^160^–^162^ Among studies that examined average daily alcohol use, results were less clear when examining various facets of cognitive functioning. One study found a U-shaped association between the number of drinks per day and dementia risk over 7 years among older men and women age 65 and older, with lowest risk for those consuming about three drinks per day; however, the authors did not differentiate between people who never drank or who quit drinking or reasons for abstinence.^163^ Similarly, two studies showed that older women who did not drink had poorer cognitive functioning compared to women who drank less than one drink per day; one of these studies tested mental status and memory,^164^ and the other tested global cognitive function and executive functioning.^165^ The extent to which these findings have greater implications for abstinent women than for other groups of women who drink is unclear. Additionally, there may be confounding effects of prior level of intelligence and socioeconomic status; when entered as covariates in another study, these factors were found to attenuate a positive association between alcohol use and cognitive assessments of memory and verbal ability.^166^ Another study found that women age 72 and older who consumed one to seven drinks per week (i.e., within the limits of the *Dietary Guidelines for Americans, 2020–2025*) had lower dementia risk than women who drank higher quantities, but only among those without mild cognitive impairment.^167^ Due to the low number of studies, these articles cover both dementia risk and several measures of cognitive functioning, precluding solid conclusions. AUD, however, does associate with higher risk of dementia as indicated above.

A meta-analysis of 15 studies examined alcohol use and dementia risk in adults age 60 and older.^168^ Models that only included women who were currently drinking found no association between alcohol use and dementia. Results varied by country and continent, however. For example, in the United States, older women who consumed 0.1 to 1.8 drinks per day had a higher risk of dementia compared to those who drank minimally (up to 0.02 drinks per day); studies from other continents showed lower risk of dementia with increasing alcohol use (Europe, Oceania) or no association at all (Asia) between drinking and dementia risk. The authors did not provide details regarding the component study methods or samples that would account for these differences across countries.

Only one study identified for this review examined brain volume and function in relation to alcohol use and provided results interpretable for older women specifically. It found that higher alcohol use was linearly associated with lower brain volume among older women (and men).^169^ Lower brain volume was present even among individuals drinking 0.6 drinks per day. The authors noted that if they had included information about past AUD, results may have differed, based on nuanced trajectories of brain changes during AUD recovery.

#### Additional background and summary

The literature on alcohol, cognition, and neuropsychology in older women should be understood in the context of a larger, age-neutral literature on sex differences in the effects of heavy alcohol use and AUD on cognitive outcomes. Recent narrative reviews have provided excellent overviews of this age-neutral research in women.^170^–^172^ An age-neutral review indicated that women and men with AUD commonly had reduced brain volume, compromised white matter integrity, and alterations in underlying neural activity.^170^ Unfortunately, as a whole, female participants were underrepresented in these studies, with insufficient sample sizes to permit a meaningful discussion of methods.^170^ Fama and colleagues focused on alcohol’s unique effects on cognition in adult women and included some discussion of age effects.^171^ The authors additionally discussed factors that influence research outcomes and should be considered in this research, including differences in task demands and important covariates such as age, education, socioeconomic status, depression/anxiety symptoms, and hormonal differences. Nixon and colleagues^170^ and Fama and colleauges^171^ also covered the acute effects of alcohol consumption on cognition, with Nixon and colleagues^170^ focusing on this research in older adults (men and women). In general, however, the literature on acute alcohol effects was excluded in the current review because of the focus on women with heavy alcohol use and/or AUD, populations who are generally ineligible for alcohol administration studies.

Aligning with the information summarized in this section above, Fama and colleagues discussed the perceived benefit of drinking one drink per day for women in relation to cognitive outcomes, when compared to those who do not drink or who drink heavily.^171^ The authors noted the importance of potentially confounding variables in this literature, such as age and socioeconomic status. Additionally, they highlighted the importance of balancing findings that insinuate benefits of infrequent drinking on cognitive functioning with the increased risk of physical consequences among older women such as those that have been discussed throughout this review (e.g., breast cancer, liver-related disease, and alcohol-related injuries).

Also, in alignment with the findings reported here, Fama and colleagues^171^ provided important information about the “telescoping effect,” whereby chronic, heavy alcohol use may have more pronounced negative effects on cognitive outcomes in adult women compared to men (also see Nixon et al., 2024^172^). For example, one key study among adult women that was outside of this review’s publication date range and age range (age range: 28 to 64, mean age 42) compared women in treatment for alcohol use to healthy female control participants.^173^ The study found deficits in the treatment group on several neuropsychological measures, most notably in the areas of visuospatial, verbal, and nonverbal working memory; gait/balance measures; and executive functioning. Although the specific domains of cognitive functioning that are affected by alcohol use in men and/or women have not been found consistently,^170^ alcohol’s global negative effects on different domains of cognition in women are a consistent finding that requires continued research, particularly in older women specifically. With regard to neurodegeneration, AUD was commonly accompanied by reductions in brain volume, compromise in white matter integrity, and alterations in underlying neural activity in both sexes.^170^ Overall, women remain underrepresented in these studies,^170^ and this scoping review identified no other articles focused on alcohol’s cognitive effects in older women.

Both Nixon and colleagues^170^ and Fama and colleagues^171^ have provided preliminary evidence for heightened effects of heavy alcohol use on social cognition and emotional processing in women compared to men and have highlighted these particular cognitive effects as areas of needed research. The age-neutral research on alcohol’s effects on cognition and neural function and structure has important implications for older women. Given that recent generations of older women are drinking alcohol at increasing rates, it is important to extend this work to focus on women age 50 or 60 and older. Additionally, with more research findings, the ability to educate older women (e.g., at medical appointments or via public service announcements) about alcohol’s cognitive effects may become a powerful motivator for older women to reduce alcohol use or abstain (see “Prevention” section below about cognitive decline being a potential motivator for reducing drinking among older women). Also, the effects of sex and heavy alcohol use/AUD may interact differently at different points throughout the lifespan,^174^ suggesting the need for research that is focused on the interactive effects of age, alcohol use, and sex. The reviews cited here^170^–^172^ highlight the lack of research focused on age as a moderator of alcohol’s effects in women with heavy alcohol use or AUD, supporting the call for additional research with this population.

### Screening, Assessment, and Treatment Needs of Older Women With Heavy Drinking and AUD

The studies retrieved for this scoping review on screening, assessment, and treatment needs of older women with heavy drinking or AUD; their main characteristics; and their main findings are summarized in Appendix 3.

#### Treatment access barriers and facilitators

No studies have addressed treatment access or barriers and facilitators of treatment for alcohol misuse among older women. The wider sex- and age-neutral literature (which was not extracted as part of the scoping review) has indicated that women with AUD face particular barriers to seeking and receiving help, and fewer women (15%) than men (23%) with AUD seek treatment for AUD in their lifetime.^15^,^175^ Women may be more likely to seek help if single-sex treatment is offered;^176^,^177^ however, separate treatments for women have been found to be more efficacious than mixed-sex treatment only if they include female-specific programming.^17^ Research also has shown that women of all ages tend to seek mental health care in primary care settings and cite stigma as a barrier; moreover, women generally are not likely to offer information to health care providers about alcohol or drug use unless asked.^15^ Additional information on research on improving screening and disclosure of alcohol use with older female patients is provided in the “Prevention” section below.

#### Screening and assessment

Six articles focused on screening and assessment of heavy drinking or AUD among older women.^63^,^178^–^182^ In a sample that included women age 60 and older who drank and those who were abstinent, a “best predictor model” of heavy drinking included using two or more over-the-counter drugs regularly, consuming large amounts of coffee, and using alcohol to fall asleep; other predictors included smoking, mixing over-the-counter drugs with alcohol, and slower sleep latency.^63^ However, the study did not account for individuals who had quit drinking and did not include enough subjects in this age group to allow for valid statistical analyses; therefore, it may have identified a specific population of women age 60 and older who drink alcohol in older age.^63^ In terms of screening measures, evidence was found for better performance of the AUDIT, compared to the Short Michigan Alcoholism Test Geriatric Version (SMAST-G), in identifying women age 65 and older who drank heavily (i.e., eight or more U.S. standard drinks per week).^156^ The AUDIT-C also outperformed the Comorbidity Alcohol Risk Evaluation Tool (CARET, another measure adapted for older adults) in a sample of women age 50 and older.^157^ Specifically, the AUDIT-C was more sensitive and conservative compared to the CARET, which is also more complex and time consuming to administer.

Two of the six articles addressed the reliable and valid use of the DSM to identify AUD among older women.^180^,^183^ One study found that women age 55 and older first met DSM-IV AUD criteria 5 to 6 years later than men.^180^ Moreover, 77% of older women reported patterns of symptom onset that were not in line with general population models of AUD symptom onset and progression. For example, these older women with alcohol misuse were more likely than older men with alcohol misuse to report drinking despite health problems and less likely to report use in hazardous situations (i.e., in situations in which it was dangerous to drink) or alcohol-related interpersonal problems (i.e., experiencing problems with family members or friends or getting into physical fights as a result of drinking). A more recent analysis of specific DSM-V AUD criteria showed that women age 50 and older were less likely than older men to endorse drinking larger amounts than intended, having legal problems, or not meeting role obligations.^181^ However, women at the lower end of the alcohol dependence severity spectrum were more likely than older men to endorse drinking larger amounts than intended (loss of control), which is atypical for a progression model.

These findings suggest that older adults (both men and women) who are at risk for alcohol-related health problems remain under-identified and that DSM classification alone is not sufficient.^181^ Instead of screening only based on consequences of use or classifications of risk, it may be helpful to also assess frequency and quantity use patterns to identify alcohol misuse among older women. Thus, one study of adults ages 21 to 70 found higher frequency and lower quantity of consumption among older adults compared to younger adults.^182^ Moreover, consuming more than three drinks on any day or eight drinks per week was associated with greater risk for alcohol-related problems among the older, compared to younger, age groups of women.

##### Summary

The results of the studies reviewed here overlap with sex-neutral AUD screening and assessment recommendations for older adults in general, which include use of the AUDIT with age-sensitive scoring.^184^ Stigma-sensitive screening may be especially important for older women^184^ as would be widespread screening in places where older adults are typically seen for medical, psychiatric, and social services care.^184^ Under-identification of alcohol-related problems among older women and men^181^ may be ameliorated through accessible online screening and referral tools, such as the NIAAA Rethinking Drinking website (https://rethinkingdrinking.niaaa.nih.gov/) and the NIAAA Alcohol Treatment Navigator (https://alcoholtreatment.niaaa.nih.gov/). Similarly, NIAAA has online resources such as the NIAAA Core Resource on Alcohol (https://www.niaaa.nih.gov/health-professionals-communities/core-resource-on-alcohol) to help facilitate widespread training of medical and mental health professionals in addiction and alcohol/drug screening and referral. With regard to screening and assessment, it is also important to note findings that women age 50 and older are more likely than their male counterparts to understate the harm of their personal drinking, even when they are aware of what constitutes heavy drinking or high-risk levels of alcohol use.^185^

#### Unique treatment needs of older women with alcohol misuse

##### Triggers, high-risk situations, relapse antecedents, and reasons for using alcohol

Fifteen articles provided information that was relevant for identifying unique treatment needs of older women (note that many of these have also been covered in the “Correlates” sections above).^58^,^61^,^72^,^78^,^82^,^87^,^91^,^94^,^95^,^97^,^101^,^102^,^186^–^188^ Factors that were associated with either alcohol use or AUD symptoms among older women included tension reduction motives and social networks that approve of drinking,^97^ co-occurring mood^78^,^82^ and anxiety disorders,^78^ risk of later development of anxiety and depression,^87^ and exposure to emotional and physical abuse.^78^ Additionally, older women might be more likely to drink in response to negative affect (e.g., stress,^188^ anxiety,^78^ distress,^61^,^72^ and loneliness^186^) than older men, although more research is needed. Some studies compared women to men with respect to putative reasons for drinking (including mood and anxiety disorders) and found that these associations were statistically more likely to be present for women than for men,^61^,^72^,^78^,^82^,^87^ with just one study finding these associations among both men and women.^97^ Studies included both nonclinical and clinical samples of older women, including some seeking treatment. Although this work suggests that the likelihood of drinking to cope with, or in the context of, negative affect may be sex-specific in older age, continued research is needed.

One study compared stress exposure of men and women ages 62 to 78 who endorsed alcohol-related problems (i.e., at least one DSM-5 AUD symptom) to those who drank alcohol but did not report any problems.^188^ Women with alcohol-related problems reported more exposure to certain types of stressors (e.g., partner drinking; family interpersonal problems such as problems with children, spouse, or other family members; recent death of someone close; general emotional distress) than their male counterparts, but did not differ from women without alcohol-related problems. However, the women reporting problems related to their drinking were more likely to drink alcohol in response to these stressors than women without alcohol-related problems (i.e., were more likely to engage in drinking to cope).^188^

Older women, like women of any age, tend to engage in alcohol use more at home and alone.^186^ However, this may change with the baby boomer generation, which is more tolerant of heavy drinking and cannabis use in social situations.^186^

The results described thus far regarding factors related to alcohol use can also be found in the age-neutral literature on sex differences in alcohol use and AUD. In addition to alcohol-related treatment needs that women of any age experience, additional factors that contribute to potential age-specific treatment needs include retirement,^95^ physical pain,^72^,^187^ and fatigue.^72^ The studies assessing these factors compared older men and women, and those that included heavy-drinking and/or treatment-seeking samples (rather than nonclinical samples)^72^,^95^ found that these factors were associated with drinking among women but not men. Women age 60 and older in treatment for AUD, compared to their male counterparts, may be at higher risk of “loss of control” drinking, including experiencing irresistible cravings, drinking more than they intended, and continued alcohol use despite consequences.^91^ All of these risks can be targeted with specific interventions or medications.

Additional specific treatment needs may relate to a potential need to treat use of multiple substances. Women age 65 and older who engage in binge drinking are more likely to endorse cannabis use than those who do not binge.^58^ Furthermore, AUD in older men and women is highly comorbid with tobacco use disorder.^82^

Finally, heavy drinking and alcohol-related consequences are more common among older women who are married than among unmarried women, including those who are divorced, widowed, or never married.^61^,^94^,^97^,^101^,^102^ This finding may have implications for treatment approaches within this population and potential couple- or family-based interventions.

##### Summary

Evidence for several potential triggers, high-risk situations, relapse antecedents, and drinking motives among older women has replicated findings in the wider age-neutral literature on women and alcohol.^17^ Specifically, these include findings related to drinking in the context of negative affect, the link between drinking and marital status, and the likelihood of drinking alone and at home. While some of the female-specific treatment needs outlined here are age-neutral, this scoping review identified no research that compared older to younger women. Therefore, it is difficult to know if any treatment needs are age-specific in addition to being sex-specific. For instance, whether the associations found between marital status and alcohol use/AUD risk are different, or more common, among older women compared to younger women has not been studied. Differences among age groups of women with heavy alcohol use and AUD may have implications for prevention and treatment approaches and may also indicate naturally occurring developmental transitions in alcohol-related habits across women’s adult lifespan. Much of this research is also correlational and based on self-report; although this is expected to some extent, work that uses alternative methodology such as ecological momentary assessment or behavioral tracking may advance understanding of how and why older women drink on a daily basis.

#### Use and misuse of prescribed medications and cannabis

Studies have found older women who drink alcohol were more likely to use medications with addictive potential compared to men who drink.^99^,^189^,^190^ In a female-only sample of women age 50 and older (53% Black; 46% with food insecurity; 27% employed), 30% reported both prescription opioid use and binge drinking in the prior 30 days.^190^ Women who reported prescription opioid use, with or without binge drinking, were more likely to also report back pain, cancer, depression, and anxiety. In another analysis, women who reported drinking on one or more days per week were more likely than men to use drugs with addiction potential (benzodiazepines, nonbenzodiazepine hypnotics, or opioids).^189^ Similarly, older women with binge drinking were more likely to engage in nonmedical use of prescription drugs compared to women who consumed alcohol but did not binge drink and those who did not drink at all; again, these differences were not observed among men.^99^ With respect to over-the-counter medications, women over age 60 who used relatively more of these medications were more likely to drink alcohol than to be abstinent.^63^ In a study across 65 primary care clinics, 25% of female patients at age 65 and older were found to be engaging in “risky” alcohol use (defined as consuming alcohol in combination with alcohol-interactive medications, drinking despite contraindicated medical conditions, and/or other alcohol-related behaviors such as drinking and driving); however, it was unclear how many of those women were specifically in the interactive medication use category versus other risky drinking behaviors.^191^

With respect to cannabis use, a national survey found that women age 65 and older who engaged in binge drinking in the past month were more likely to use cannabis compared to women who did not binge drink (in separate analyses, the same was found for men of this age group).^58^

##### Summary

In the wider literature, studies have highlighted the comorbidity of addictive medication use and risky drinking among older women.^192^ These studies on co-occurring alcohol use and other substance use among older women had notable limitations, including reliance on self-report measures and reliance on secondary use of datasets. The latter factor may lead to convenience samples that limit generalizability (e.g., having small samples of women with heavy alcohol use or AUD, specific racial/ethnic representation, samples of only primary care patients). Additionally, most of the studies did not account for lifetime alcohol use patterns; thus, some participants who misused prescription medications in older age may have been more likely to have histories of alcohol misuse. These limitations may lead to underestimations of rates of concomitant use. Additionally, these studies did not go beyond establishing rates and prevalence. For example, there was no research on psychosocial differences between older women with and without co-occurring substance use, which would have important treatment and prevention implications.

#### Treatment needs of subpopulations of women

A few articles assessed subpopulations of women age 50 and older with AUD, such as older women from different racial/ethnic backgrounds^190^,^193^ or lesbian older women,^104^,^194^,^195^ with most of these studies providing information on drinking patterns and rates of drinking within these specific subpopulations. Collectively, these studies tended to have small sample sizes and relied on recent alcohol use (past 30 days) only. Qualitative studies might be particularly helpful to guide treatment development for specific subpopulations of women while this area of research is developed. For instance, interviews of 20 lesbian women ages 50 to 70 with DSM-IV alcohol abuse/dependence highlighted helpful aspects of treatment entry and treatment programs, such as engagement in formal treatment plus 12-step programs, helpfulness of nonfamilial community-based sources (e.g., employer, health care provider, legal system, religious or school system), and the significance of “wake up calls” from supportive significant others about high-risk alcohol use.^195^ Participants also reported high levels of resilience that allowed them to “bounce back” after adverse situations. This latter element of self-efficacy could be particularly salient for subgroups of women in older age.

### Alcohol Treatments for Older Women

Currently, there are no age-tailored, female-specific treatments or interventions for older women with heavy alcohol use or AUD. Promising sex-neutral treatments for older adults have been tested (many of which include female participants),^183^ as have age-neutral treatments for female adults.^31^ Existing sex-neutral treatments for older adults (which were not captured in this scoping review) have been reviewed by Kok^196^ and Kuerbis and Sacco.^183^

The studies retrieved for this scoping review on alcohol treatments for older women, their main characteristics, and their main findings are summarized in Appendix 4.

#### Older women in sex-neutral AUD treatments for older adults

Eight studies examined the outcomes of older women in sex-neutral treatment trials with men and women.^197^–^204^ Of these, about half were secondary analyses of parent studies, including four from the same research team and site.^197^–^199^,^204^ In that series of secondary data analyses, the authors compared outcomes for older adult men and women (age 55 and older) in sex-neutral outpatient treatment at a community clinic. At 6 months^204^ and 7 years^198^ posttreatment, older women reported higher rates of abstinence versus men, with similar results for 5-year follow-up in a related study.^197^ Women ages 55 to 77 (mean age of 60) stayed in treatment longer than their male counterparts did,^204^ and more time in treatment predicted better outcomes at 6-month and 7-year follow-ups for all participants.^198^,^204^ Across 5-, 7-, and 9-year follow-up points, with no sex differences, adults age 55 and older (and middle aged adults ages 40 to 54) were more likely than those ages 18 to 39 to be abstinent from alcohol.^199^ Moreover, female sex (compared to male sex) at any age was associated with higher rates of remission from alcohol-related problems across years 5 to 9. Other factors associated with remission from alcohol-related problems in older adults (with no sex differences) across years 5 to 9 included not losing a partner to separation, divorce, or death; not experiencing a decline in health; having any close friends supportive of recovery; and not having any close friends who encourage alcohol or other drug use.^201^ In sum, the results from this set of analyses suggest favorable long-term prognosis of older women in a general outpatient treatment clinic.^199^

Two randomized controlled trials (RCTs) specifically tested psychosocial treatments for older men and women.^200^,^201^ One study conducted a feasibility testing of an online normative feedback intervention versus personalized feedback brief interventions for people age 50 and older who were drinking.^200^ The normative feedback intervention provided information about participants’ drinking compared to their peers of the same age and sex; the personalized feedback intervention provided structured feedback for individual participants regarding suggested drinking limits and typical drinking patterns of their peers. No sex differences in outcomes were found. Both female and male participants underestimated their drinking risk at baseline: 80% of participants self-reported as no- or low-risk drinkers, but 52% were actually at-risk drinkers according to the CARET assessment (based on alcohol consumption, risk behavior like drinking and driving, and drinking in combination with contraindicated medical conditions/medications, see Table 1). Overall, participants found the intervention feedback helpful and reported a preference for online versus in-person intervention. Moreover, 44% of participants made a plan to change their drinking behavior, with participants in the normative feedback group more likely to make a change plan than those in the personalized feedback condition.^200^ A multinational RCT^201^ testing motivational enhancement therapy plus a “Community Reinforcement for Seniors Approach” versus motivational enhancement alone for men and women over age 60 yielded promising results for alcohol abstinence and quality of life for both men and women. Motivational enhancement aims to increase patients’ internal motivation to change their alcohol use behavior. Community reinforcement involved several levels of cognitive behavioral and social network interventions to promote reductions in alcohol use. There were no treatment condition differences in outcomes based on sex, although older women were less likely than older men to achieve certain successful outcomes (i.e., having a blood alcohol content ≤ .05% in the 30 days before assessment) at 26 weeks postbaseline.^202^ Quality of life and changes in drinking were also examined by participant sex.^202^ Across both treatment conditions, both men and women reported improved quality of life in the physical, psychological, social relationships, and environmental health domains.

Other investigators compared differences in treatment response among men and women ages 50 to 88 based on participants’ self-reported age at which they started to have “alcohol problems.” The term was not defined in the article, but participants had an average AUDIT score over 19.^203^ Participants were enrolled in a U.K.-based program for men and women over age 50 with “alcohol problems,” in which they were provided with age-sensitive assessments (e.g., screening for cognitive impairment, fall risk, elder abuse, alcohol-medication interactions), interventions adapted for cognitive impairment and focused on life-stage issues, and peer support groups. Age of onset in this older adult sample (i.e., < 25, 25 to 39, 40 to 59, or ≥ 60 years old at time of onset) was unrelated to participant sex or to treatment outcome; however, women decreased their use of alcohol following treatment more than men.^203^

#### Older women in age-neutral, female-specific AUD treatments

A secondary analysis of an earlier RCT compared the female-specific, women-only Early Treatment for Women with Alcohol Addiction (EWA) with mixed-sex treatment as usual (TAU).^205^ The EWA treatment protocol comprised individualized options across a continuum of care, from detoxification to individual and group sessions, and ongoing contact for 2 years. TAU included regular contact with nursing staff and prescription of disulfiram to patients after they completed inpatient or outpatient detoxification. The analysis assessed mortality rates of women age 71 and older at 27 years after treatment (average age at baseline was 42). At 27-year follow-up, lower mortality rates were found for women in the more intensive, women-only EWA versus less intensive, mixed-sex TAU, regardless of their age at intake. However, effects were stronger among women who began the program at a relatively younger age.

Another secondary data analysis used pooled data from two RCTs among women with DSM-IV alcohol dependence.^206^ The study compared participants in three age categories (> 55, 45 to 55, < 45) on baseline variables and on response to cognitive behavioral treatment/motivational enhancement treatment. Treatments were provided in weekly couple or individual sessions, using sex-neutral or female-specific protocols. At baseline, prior to treatment, women older than age 55 had better psychosocial functioning, more supportive social networks, less severe lifetime substance use history, but more frequent and heavy drinking compared to women under age 45 and those ages 45 to 55. Moreover, compared to the younger age groups, women older than age 55 were more engaged with treatment and, at 1-year posttreatment, showed greater reductions in their number of drinking and heavy drinking days.^206^

##### Summary

Existing female-specific treatment protocols^15^,^207^ that are efficacious for women of all ages may also be particularly useful for treatment of older women. However, this literature is very small and requires further research. Female-specific therapies might be tested for older women versus older men, and/or in a telehealth format. Efficacious interventions for women may be further researched and developed by delivering and disseminating them in medical and mental health settings where older women with AUD are likely to receive care.^31^

#### Untreated remission

Remission without receiving formal treatment was assessed in one secondary analysis of a 10-year study of drinking course among 578 men and women ages 55 to 65.^208^ All participants reported at least weekly alcohol use at baseline. Compared with people in remission who had received treatment and those who had not received treatment and were not in remission, (i.e., treated remitters and untreated nonremitters), individuals in remission who had not received treatment were more likely to be women. Women who were advised by family, friends, or others to reduce drinking attained remission more often than men who received advice.^208^

##### Summary

This study has implications for development of brief interventions for older women that involve significant others (i.e., family or friends). Although only one such study was included in this review, it echoes findings described above indicating the relevance of relationships in older women’s drinking patterns,^58^,^96^,^99^,^195^ as well as findings indicating that women are more likely to quit drinking with age.^31^,^93^,^94^,^97^ Of note, and relevant to the topic of remission without formal treatment, no studies were found on the unique needs or outcomes among older women in Alcoholics Anonymous or other self-help groups. Peer support in such groups, especially if available in a hybrid in-person and online format, might be particularly helpful and acceptable for older women.

#### Pharmacological interventions

This review identified one RCT testing a pharmacological intervention in older adults since 2004.^209^ The RCT tested the impact of treatment with naltrexone + sertraline + psychosocial support versus sertraline + psychosocial support (no naltrexone) on alcohol consumption and depression. Participants were 74 men and women over age 55 who were diagnosed with co-occurring DSM-IV depressive disorder and alcohol dependence. The study assessed “overall response to treatment,” which was defined as remission in depression and absence of relapse to binge drinking. This overall response was achieved by 72% of women treated with just sertraline + psychosocial support, compared to 25% of women treated with naltrexone + sertraline + psychosocial support.^209^ The lower percentage of improvement in the latter condition was attributable to lesser improvement in depression, not binge drinking. Results must be interpreted with caution, however, because there were only 15 women in the sample, and the primary outcome variable “treatment response” focused on absence of binge drinking as the only alcohol-related outcome.^210^

### Prevention of Alcohol Misuse and AUD in Older Women

The literature on prevention of heavy drinking or AUD (primary, secondary, or tertiary) is limited for older women. The lack of alcohol use screening with this population is highlighted among the articles that were identified in this review.^211^,^212^ For instance, among 5,000 women age 65 and older, 27% who used alcohol reported not being asked about their drinking in any prior-year health care encounter, and older women were less likely than men to report having discussed alcohol use with any providers.^211^ One RCT compared a computer-delivered animated brief alcohol intervention (“Health4Her”) + lifestyle health prevention to the lifestyle health promotion only for women attending routine breast screening.^213^ Outcomes at 12-week follow-up showed improvements in understanding of alcohol as a risk factor for breast cancer and in alcohol literacy, as well as decreased alcohol use in the Health4Her condition.

Relatedly, there is evidence that older women’s decisions about drinking may be made within a larger lifestyle context that includes decisions about diet and exercise.^214^ Several correlational articles included in this review revealed complicated associations between health burden, health perception, and drinking risk, with implications for prevention and treatment in the general population of older women (i.e., among those without AUD).^57^,^60^,^61^,^94^,^96^,^101^,^108^,^215^ In general, among older women, self-perception of better physical health may be associated with alcohol use rather than abstinence, and perception of poorer physical health may be associated with decisions not to drink or to drink less when compared to women who view themselves as healthy. As described above, several studies found that worse perceived health was associated with lower likelihood of drinking or heavier drinking among older women:

- Better self-reported physical health was associated with heavy drinking (≥ 7 drinks per week).^57^
- Women with “fair” or “poor” health were less likely to report drinking problems compared to women with good health.^215^
- Illness or disability prior to retirement was associated with a lower likelihood of any drinking.^57^
- Women who reported declines in physical health across a 10-year period were more likely to reduce their drinking than women who experienced consistently good or poor health.^94^
- Women with self-perceived poor health drank less frequently than women with self-perceived good health across 3 to 6 years.^101^

Although none of these studies accounted for lifetime alcohol use, two other studies that did found similar—albeit, slightly different—results. Specifically, self-rated health was positively associated with alcohol consumption, but only up to two drinks per day, among older women.^60^,^108^ Moreover, the positive association between perceived health and alcohol use diminished with increasing alcohol use.^60^ In another study, better self-reported health was associated with higher likelihood of alcohol misuse (defined as an AUDIT-C score ≥ 3, based on quantity/frequency of drinking), but a lower likelihood of reporting problems or consequences from drinking on the AUDIT questions.^61^ These latter studies suggest that better health may be associated with the decision to drink any alcohol and with quantity and/or frequency of alcohol use, but is not associated with very heavy alcohol use or alcohol-related problems. In other words, older women in the general population (not necessarily those at risk for AUD) who perceive themselves as healthy may be more likely to drink alcohol, but not necessarily to excess. Additionally, women who are in relatively poor health and still drink alcohol (but not those who are in poor health and therefore decide not to drink) may be more likely to experience consequences related to their alcohol use and/or have less resources to deal with alcohol-related consequences, increasing the likelihood that they may meet criteria for an AUD. Further research to enhance understanding of these associations between health and alcohol use will be important for developing tools for screening, prevention, and treatment of alcohol-related problems among older women.

Qualitative and implementation science methods may be helpful for developing prevention messaging and interventions for subpopulations such as older women. One study conducted focus groups with women ages 40 to 65 and with expert stakeholders to develop a prevention intervention regarding alcohol consumption and breast cancer risk in this age group.^136^ Psychological capability (i.e., knowledge), social opportunity (i.e., social pressure), and automatic motivation (i.e., drinking to cope) were identified as barriers to behavior change that could be targeted in an intervention for older women. Suggestions were to tailor information to women’s experiences, address the perceived social benefits of drinking (e.g., acknowledge that some women view drinking as routine or integral to their social interactions), and teach healthy coping strategies; also, acceptable messaging should not be judgmental or patronizing, and may include personal stories, simple statistics, and healthy alternatives.^136^ Research on acceptability of alcohol-related messaging in health care settings is important because of evidence that women may be more likely than men to follow alcohol-related advice generally (e.g., by family, friends, or others)^208^ and in relation to specific health risks. For instance, women age 50 and older reported being likely to follow drinking guidelines if that would lower their risk of developing dementia.^216^

Furthermore, women in general have inaccurate beliefs about the health value of wine and about drinking guidelines.^185^,^212^ Women age 50 and older were more likely than their male counterparts to overestimate or not know the level of alcohol consumption that was associated with health risks; moreover, even when they did correctly estimate heavy drinking or high-risk levels, they understated the harm of their personal drinking.^185^ Raising awareness of guidelines for older adults is especially promising for older women who tend to be receptive to alcohol-related health conversations with health care providers, particularly about alcohol–medication interactions.^212^ Although, as mentioned above, older women are unlikely to spontaneously bring up alcohol use as a primary concern,^7^ this literature suggests they are open to conversation and advice if initiated by a provider.

#### Summary

Other than drug labeling, no interventions are currently being widely implemented to prevent adverse medication–alcohol interactions in older women (including using addictive medications not as prescribed), despite high prevalence rates of these behaviors in older men and women.^217^ A study that was not captured in the scoping review because its results were not stratified by sex tested a brief educational primary prevention intervention (i.e., informational poster, brochure and brief video service announcement based in a health belief model and an information-motivation behavioral skills model) for men and women over age 60.^217^ Initial results showed a reduction in alcohol use by heavy drinkers and sustained change in attitudes toward medication–alcohol interactions. This study represents a promising educational campaign for preventing adverse events related to heavy alcohol use in older adults. Sex-stratified analyses and results would support greater understanding of how to best prevent alcohol risk and alcohol-related problems in older women.

Based on this prevention literature and on the alcohol-related consequences that have been studied among women, another important prevention strategy may be to raise awareness of the personal risk factors that contraindicate any drinking at all for older women. This could include educational campaigns as well as primary screening and secondary prevention interventions (including screening, brief intervention, and referral to treatment, or SBIRT, intervention models) to increase the knowledge base on older women’s risks of heavy alcohol use, including such topics as how biological aging can accelerate and aggravate level of intoxication and medical consequences for older women.^37^ Thus, primary prevention educational campaigns and interventions might highlight risks of alcohol use and benefits of abstinence or alcohol use within the recommendations of the *Dietary Guidelines for Americans, 2020–2025* in the context of healthy lifestyle habits and successful aging.

## Discussion

### Summary of Evidence

After age 50, women negotiate multiple milestones and lifespan transition events as they age—including, but not limited to, menopause, retirement of self or spouse, widowhood, isolation, care of elderly parents, shrinking social network, fixed income, and health decline—that can contribute to risk and maintenance of alcohol misuse. The studies reviewed here suggest that women struggling with depression,^78^,^81^,^83^,^87^,^88^ isolation,^95^,^103^ and trauma and chronic stress^76^,^84^ may continue to be at higher risk for alcohol misuse as they progress into older age. Additionally, social factors such as significant others,^94^ quality of social support,^103^ socioeconomic factors,^108^ and overall health^59^–^61^ all influence women’s drinking in older age. As described in the “Alcohol and Cancer”^110^ section, research has established alcohol use as a risk factor for certain types of cancer, with the most evidence on increased breast cancer risk. However, more quality research is needed on other physical and psychological consequences of drinking in this population. In terms of treatment, older women were found to have better outcomes than older men in some cases^197^,^198^,^202^,^203^ but not in others.^201^ Furthermore, research suggests that women are more willing than men to listen to advice regarding risks of drinking^212^,^216^ and that they respond well to brief interventions with online normative or personalized feedback.^200^ Collectively, this research suggests that older women can benefit from prevention and treatment efforts aimed at reducing heavy alcohol use, AUD, and dangerous alcohol use (e.g., mixing drinking with prescribed medications), and continued research on this population will help improve women’s health.

Women (and men) must be made aware by medical providers and public health messaging that, with age, women should revisit and reevaluate their frequency and quantity of drinking.^185^,^212^ Although research is limited in all areas covered by this review and many questions remain, a clear picture emerges in which heavy alcohol use conveys significant biopsychosocial consequences particularly for older women. Women with heightened risk (e.g., those with prior alcohol misuse, a family history of AUD, heavy drinking partners or social networks, chronic health conditions, or mood disorders) would especially benefit from such information. Physiological changes that are associated with aging and are aggravated by alcohol use can increase the likelihood of medical conditions, health decline, chronic pain, insomnia, and depression in older women, which in turn can further trigger more alcohol use.^61^–^63^,^72^,^87^,^88^,^187^ In sum, difficulties that can arise with aging in some women can compound alcohol-related risk, and alcohol misuse impedes successful aging among this population.

### Gaps in Research

Most of the literature on older women identified in this scoping review focused on physical consequences of alcohol use, with an over-representation of large-scale survey and/or epidemiology studies conducted primarily with community (i.e., nontreatment-seeking) male and female populations. There is a dearth of treatment research for older women misusing alcohol. Only 30 articles with any relevance to treatment of alcohol misuse among older women were found. Eleven articles addressed efficacy of existing treatments available for older women. Among these, only two were primary source RCTs that developed and tested new treatments for older adults with AUD and presented sex interactions.^200^,^201^ No RCTs to develop new treatments for older women with AUD or heavy drinking were found. Most AUD treatment studies (RCT or other) did not analyze data specifically to understand effects for older women (i.e., did not stratify analyses by sex and/or older age, and/or did not show data separately for older women versus older men or younger women). Indeed, 738 articles were excluded from the review for failing to examine sex differences and/or age-by-sex analyses, which is not aligned with NIH policies (described in the introduction) that require such analyses (especially on sex differences) in clinical trials. Missed opportunities for stratified age-by-sex analyses to yield important information on older women were plentiful. Most studies (treatment or nontreatment) included very small samples of older women with heavy drinking or AUD. Research on prevention development for heavy drinking or AUD among older women was almost nonexistent and is crucial to stemming the current burgeoning public health crisis of excessive drinking among older women. The nontreatment research literature also had notable gaps. For several relevant topics, no or very few articles were found, including alcohol misuse in relation to retirement, research on subpopulations of older women (e.g., women from any type of minority racial or ethnic background), gastrointestinal consequences of alcohol misuse, and the association of brain structure and function with alcohol misuse specifically in older women (note that important work on this topic is done in mixed-sex samples, as described in the “Cognitive and Neuropsychological Consequences” section above, but the findings did not meet criteria for the current review). Other gaps included alcohol metabolism and acute effects after menopause, menopause as a transitional phase in relation to AUD risk, the impact of loss and grief, quality of life, stigma and social norms, and long-term recovery.

As described in the introduction, menopause may be a critical life transition phase in relation to understanding women’s alcohol use and their alcohol-related risk for physical and psychological conditions. There is very limited research on these topics. Although disentangling age- and menopause-related effects is a complicated task, it is critical to understanding older women’s risk in relation to alcohol consumption.

An additional gap in research are studies that would take a more nuanced approach to studying “younger older women” versus “older older women.” This review’s definition of older women (i.e., age 50 and older) potentially covers more than 40 years of life. In younger individuals, alcohol use and AUD differ in very important ways between adolescence, early adulthood, college years, and the 30- to 40-year-old range. These differences have impacted the way that prevention and treatment are approached for these age groups.^218^ It is just as likely that differences with important treatment implications would emerge for adults over age 50. Even within the large survey studies included in this review, it was the exception rather than the rule that such age groups were defined and analyzed. In the appendices summarizing data extraction for the various topics, the “Age Differences” column notes whether the study compared age groups or analyzed age as a continuous variable in relation to outcomes. Notably, when age groups were tested, differences were often found. For example, in the age group 55 to 64, women with heavy drinking were more likely to report distress due to pain, sleep, and fatigue than their male counterparts; however, these sex differences were not present in the age group 65 to 74.^72^

### Methodological Issues

There are significant methodological issues in the existing literature that can have misleading and potentially harmful implications, such as inconsistent or ill-defined drinking terminology, lack of consideration of alcohol use history, or small sample sizes. As detailed in the “Methods” section, this review excluded most of the literature where results could not be reliably interpreted because of such methodological issues or in which authors provided insufficient information about their methodology to make such a determination. However, it is important to note these limitations here because of their impact on the amount of research findings available on these topics. Identifying these issues may also help facilitate sound research on alcohol and aging in the future.

One concern was inconsistent and sometimes inaccurate use of terminology related to drinking. Terms such as “moderate drinking,” “risky drinking,” “heavy drinking,” and “alcohol problems” were commonly used, without aligning with official definitions provided by NIAAA, WHO, the National Cancer Institute, or any other health organization. While these terms may seem relatively innocuous, they could lead to highly divergent and thus inaccurate results; for example, definitions of “moderate drinking” ranged from 0.5 drinks per day to three drinks per day or more across studies. For articles retained for this manuscript, only standardized terminology as defined in the “Notes on Terminology and Definitions” section and standardized drinks aligning with NIAAA definitions were used. Thus, when a study used “moderate drinking” to refer to no more than three U.S. standard drinks per day, the number of drinks was provided here rather than vague terminology. However, this was not always possible and/or clearly explained by some papers, which were excluded as a result. Precise and consistent terminology in relation to alcohol use is critical because it is difficult for health care professionals and public audiences to make sense of these findings with inconsistent use of terminology.

As with the general aging and health literature, the treatment of “sick quitters” (i.e., individuals who quit drinking in later life due to health decline, whether alcohol-related or not) was a common concern in interpreting study results. The “Quitters” column in the appendices with the extraction tables indicates whether each study accounted for former drinking in assessment and analyses. Some studies separated “former drinkers” from “never drinkers” in their analyses. However, many studies did not attend to this distinction in their analyses, including articles retained and extracted in this manuscript. Similarly, socioeconomic status and related variables emerged as important covariates in research with older women and are important to account for in research moving forward.

Another concern is research that may be biased toward supporting “moderate” drinking as being healthy. This scoping review on methodologically sound studies did not yield any evidence for health benefits of alcohol consumption, especially when considered in balance with the various health risks of drinking. In addition, most studies compared “low-risk drinking” (loosely defined) with no drinking, rather than comparing individuals with heavy drinking or AUD with those who never drank or who drank within guidelines (e.g., the *Dietary Guidelines for Americans, 2020–2025*) throughout life, to examine alcohol-related consequences. Another common error was ignoring survivor bias. Because heavy drinking and AUD are associated with higher mortality, one can expect that samples of older adults who drink might not include individuals who were most negatively affected by alcohol use (e.g., who had become very sick or had died prematurely and therefore were unable to participate). Additionally, any findings suggesting that occasional drinking may be correlated with any sort of positive outcome must be weighed with the evidence of alcohol’s negative effects—for example, that even small amounts of drinking were associated with increased cancer risk or decreased brain volume.

Many articles were excluded from the review because they included very low numbers of women who drank more than occasionally (defined as drinking less than monthly on average) or who drank any alcohol at all. Despite those small sample sizes of the target population, some of these studies conducted sex analyses and reported results, raising concerns about statistical validity or lack of power to find any possible associations. Relatedly, much of this literature was based on epidemiological cohort studies. Although large-scale research can provide critical information about prevalence and health risks, these methods are not ideal for targeted recruitment of women at risk of alcohol misuse/AUD, for using reliable and valid measurements of alcohol use, or for AUD diagnoses. This has resulted in a lack of research among women with clinical levels of alcohol misuse (binge drinking, consistent heavy drinking, or AUD). The highest drinking category in many studies was one to two drinks per day, and many studies eliminated from this review focused on samples drinking at substantially lower rates (e.g., 4 grams of alcohol, corresponding to 0.3 U.S. standard drinks, per day) or compared complete abstention to any alcohol use.

Lastly, research that examines changes in alcohol use over time has been lacking. Many of the longitudinal studies, including those on physical consequences, only assessed alcohol use at baseline in relation to eventual outcomes. Therefore, several or more years often separated the time of alcohol assessment and outcome assessment (e.g., disease onset). Given that many people fluctuate in their alcohol use across the lifespan, estimates of disease outcomes that were generated from this literature may be over- or even underestimated.

### Limitations

Careful attention should be given to the review’s inclusion and exclusion criteria and the fact that it is a scoping review, intended to provide an overview of existing literature and identify gaps in research. The review sought to focus on clinically relevant outcomes for older women, who represent a very specific subgroup of individuals with AUD. A number of steps were taken to critically analyze the research included. However, this approach may have led to exclusion of quality articles on older adults—for instance, because of the methodology used or because results could not be interpreted to generate useful information for older women. Relatedly, there is existing age-neutral research with women or sex-neutral research on older adults that has implications for older women. However, if a study’s analyses did not specifically examine older women, precluding definitive conclusions for this group, the article was excluded. Although efforts were made to describe relevant age- and/or sex-neutral research within each topic and to refer the reader to other reviews on these topics, detailed discussion of this work was beyond the scope of the review.

### Conclusions and Future Directions

Documented sex and age differences in the correlates and consequences of alcohol misuse, increasing rates of AUD among older women, and the accelerated negative impact of AUD on women’s health all underscore the need for further research on older women who drink. It is important to remember that most prevention and treatment options (including pharmacological treatments) were developed with predominantly male and/or age-neutral samples. The research presented here provides sufficient indication of differences between older women and both older men and younger women to suggest that research focused on this population is warranted. Availability of evidence-based, older–female-specific interventions is likely to increase screening and treatment utilization, as well as enhance outcomes, for older women with heavy drinking and AUD.

Primary prevention efforts may be key for older women, including information campaigns tailored to lifespan phase for women to raise awareness of alcohol-associated risks. Also, findings from this review suggest that widespread screening in places where women seek medical, psychiatric, and social services care may be a particularly effective way to reach this population. Based on existing research, implementation of personalized, detailed, stigma-sensitized, psychoeducation-laden alcohol screening starting in early adulthood and continued through mid- and late adulthood, would likely increase the probability of successful aging in relation to alcohol use and its effects. Given the increased interactions of aging women with medical providers (especially for women who drink at levels linked to development and worsening of many medical problems), primary care providers, physical therapists, gynecologists, gastroenterologists, cardiologists, neurologists, psychiatrists, and pharmacists^212^ are in a position to directly provide primary preventative education to all women patients.^95^

Findings on correlates of heavy drinking and AUD among older women also have implications for prevention and treatment development. Given the literature on social correlates of drinking, strengthening or building positive social connections and a social support network not centered around drinking may be key. In terms of co-occurring mental health conditions and AUD risk, comorbid depression, anxiety, and dysregulated stress reactivity might be addressed as triggers to use alcohol and as related treatment targets. For older women, added treatment or prevention strategies might address life transition events (e.g., loss of partner) as well as physical transition events, such as aging in general, less efficient alcohol metabolism, more pain, or menopause-related hormonal impacts on mood. Psychoeducation about the associations of drinking with healthy lifestyles, health burden trajectories, and alcohol-related risks may promote changes in alcohol misuse for older women. Similarly, clear messaging about female aging and alcohol-related risks, as well as awareness of standard drink measures and guidelines regarding alcohol consumption, may help older women to reduce drinking on their own.

## Figures and Tables

**Table 1:** Commonly Used Measures to Assess Alcohol Use and Alcohol-Related Problems

Measures	Construct Measured, Items Assessed	Scoring/Interpretation Information
Alcohol Use Disorders Identification Test (AUDIT)^21^	Assesses “harmful or hazardous alcohol use” as measured through alcohol consumption, drinking behavior, and alcohol-related problems.	Includes 10 items, with a total score of 0–40. Scores ≥ 8 are considered indicative of “harmful or hazardous” alcohol use.
AUDIT-Concise (AUDIT-C)^22^	Assesses “heavy drinking and possible alcohol abuse or dependence,” as measured through alcohol consumption only (frequency and quantity). Used as a screener.	Includes three items, with a total score of 0–12. Scores ≥ 3 are indicative of potential alcohol misuse for women (≥ 4 for men).
Drinking Problems Index (DPI)^23^	Assesses alcohol-related problems. Example items include, “felt spend too much,” “had family problems,” and “friend worried/complained about my drinking.”	Includes 17 items assessing the frequency of alcohol-related problems from “never” (0) to “often” (4). Scores range from 0–62 or 0–17 if dichotomized.
Short Michigan Alcoholism Screening Test-Geriatric Version (SMAST-G)^24^,^25^	Domains assessed include physical symptoms of alcohol dependence, drinking during emotional states (e.g., loneliness), problems moderating drinking, and others’ reactions to the individual’s drinking.	Includes 10 items, with a total score of 0–10. Scores ≥ 3 are considered indicative of “an alcohol problem,” with more comprehensive screening recommended.
Comorbidity Alcohol Risk Evaluation Tool (CARET)	Assesses “alcohol risk” for older adults via the quantity/frequency of alcohol consumption, risk behaviors (e.g., drinking and driving), others’ concerns about the individual’s drinking, medical and psychiatric comorbidities, symptoms that can be exacerbated by alcohol use, and medications which may interact with alcohol.	Includes 14 items. A participant is considered “at-risk” for each item when endorsing an amount of drinking over acceptable frequency for an age and/or gender. For example, any frequency of drinking is considered “at risk” when a participant endorses liver disease or pancreatitis.
CAGE^26^	Screener for alcohol misuse through four criteria: (1) Cutting down (desire to decrease alcohol use), (2) Annoyed (others’ critique of individual’s drinking), (3) Guilty (about drinking), and (4) Eye opener (taking a drink first thing in the morning).	Includes four items with a total score of 0–4. Scores of ≥ 2 are positive screens for AUD risk.
Addiction Severity Index (ASI)^27^	The ASI is a semistructured interview that assesses seven areas of difficulty (e.g., physical health, financial stability, alcohol/drug use) in the past 30 days and across the lifetime.	The interviewer rates each problem item on a 0–9 scale. An alcohol composite score is then created based on the number of items rated.
International Classification of Diseases (ICD-9, −10, −11)^28^	This is the official system used to assign diagnostic and procedural codes within United States hospitalizations. ICD-9 codes were used until 1999, and ICD-10 codes until 2022.	
Diagnostic and Statistical Manual for Psychiatric Disorders (DSM-IV, DSM-5)^29^	DSM–IV described two distinct disorders—alcohol abuse and alcohol dependence—with specific criteria for each. DSM–5 (2013) integrates the two DSM–IV disorders into a single disorder called alcohol use disorder (AUD) with mild, moderate, and severe subclassifications.	DSM–5 eliminated legal problems as a criterion and adds alcohol craving as a criterion for an AUD diagnosis.

**Table 2:** Strategies for Database Searches for Articles Published January 1, 1994, Through August 6, 2024

Database	Search Strategy	Hits
PubMed/Medline (including Pre-Medline and non-Medline)	(alcohol*[ti] OR drinking[ti] OR drinker*[ti] OR “problem drink*”[ti] OR “excessive drink*”[ti] OR “binge drink*”[ti] OR substance*[ti]) AND (gender*[ti] OR women*[ti] OR woman*[ti] OR female*[ti] OR “Female”[Mesh] OR sex[ti]) AND (age-adjust*[tiab] OR geriatri*[tiab] OR “old age”[tiab] OR “older adult*”[tiab] OR “older age”[tiab] OR “older female*”[tiab] OR “older patient*”[tiab] OR “older people”[tiab] OR “older person*”[tiab] OR “older woman”[tiab] OR “older women”[tiab] OR “oldest old”[tiab] OR senior*[tiab] OR “old adult*”[tiab] OR “late adult*”[tiab] OR “Menopause”[Mesh] OR menopaus*[tiab] OR postmenopaus*[tiab] OR perimenopaus*[tiab]) NOT (“Animals”[Mesh] NOT (“Humans”[Mesh] AND “Animals”[Mesh])) NOT (“Adolescent”[Mesh] OR “Child”[Mesh] NOT (“Adult”[Mesh] AND (“Adolescent”[Mesh] OR “Child”[Mesh]))) NOT (“Pregnancy”[Mesh] OR “Students”[Mesh] OR “Non-alcoholic Fatty Liver Disease”[Mesh] OR non-alcoholic[ti]) AND english[la] Limit: Publication Date: 1994/01/01 -	2,079
Embase (Elsevier)	(alcohol*:ti OR drinking:ti OR drinker*:ti OR ‘problem drink*’:ti OR ‘excessive drink*’:ti OR ‘binge drink*’:ti OR substance*:ti) AND (gender*:ti OR women*:ti OR woman*:ti OR female*:ti OR ‘female’/exp OR sex:ti) AND (age-adjust*:ti,ab,kw OR geriatri*:ti,ab,kw OR ‘old age’:ti,ab,kw OR ‘older adult*’:ti,ab,kw OR ‘older age’:ti,ab,kw OR ‘older female*’:ti,ab,kw OR ‘older patient*’:ti,ab,kw OR ‘older people’:ti,ab,kw OR ‘older person*’:ti,ab,kw OR ‘older woman’:ti,ab,kw OR ‘older women’:ti,ab,kw OR ‘oldest old’:ti,ab,kw OR senior*:ti,ab,kw OR ‘old adult*’:ti,ab,kw OR ‘late adult*’:ti,ab,kw OR ‘menopause’/de OR ‘postmenopause’/de OR menopaus*:ti,ab,kw OR postmenopaus*:ti,ab,kw OR perimenopaus*:ti,ab,kw) NOT (‘male’/exp NOT (‘male’/exp AND ‘female’/exp)) NOT (‘juvenile’/exp NOT (‘juvenile’/exp AND ‘adult’/exp)) NOT (‘conference abstract’:it OR ‘pregnancy’/exp OR ‘student’/exp OR ‘nonalcoholic fatty liver’/exp OR non-alcoholic:ti) AND [humans]/lim AND [english]/lim AND [1994–2024]/py	2,297
PsycInfo (EbscoHost)	(TI alcohol* OR TI drinking OR TI drinker* OR TI “problem drink*” OR TI “excessive drink*” OR TI “binge drink*” OR TI substance*) AND (TI gender* OR TI women* OR TI woman* OR TI female* OR TI sex) AND (TI age-adjust* OR AB age-adjust* OR TI geriatri* OR AB geriatri* OR TI “old age” OR AB “old age” OR TI “older adult*” OR AB “older adult*” OR TI “older age” OR AB “older age” OR TI “older female*” OR AB “older female*” OR TI “older patient*” OR AB “older patient*” OR TI “older people” OR AB “older people” OR TI “older person*” OR AB “older person*” OR TI “older woman” OR AB “older woman” OR TI “older women” OR AB “older women” OR TI “oldest old” OR AB “oldest old” OR TI senior* OR AB senior* OR TI “old adult*” OR AB “old adult*” OR TI “late adult*” OR AB “late adult*” OR DE “Menopause” OR TI menopaus* OR AB menopaus* OR TI postmenopaus* OR AB postmenopaus* OR TI perimenopaus* OR AB perimenopaus*) NOT (PZ Dissertation OR PT “Dissertation Abstract” OR TI nonalcoholic OR DE “Pregnancy”) AND PO Female AND PO Human AND LA English Limit To: Publication Date: 19940101–20241231	189

**Table 3:** Inclusion and Exclusion Criteria Used in the Scoping Review

Inclusion Criteria	Corresponding Exclusion Criterion [Corresponding Exclusion Number in Figure 1]
***Criteria Used During Title/Abstract Review Phase***	
Published in English	Not in English [1]
Published 01/01/2004 to 08/06/2024	Published 2003 or earlier [2]
Pertinent to older women age 50 and older	Mixed-sex older adult sample, but no sex difference analyses and no analyses stratified/conducted separately by sex [3] All female, mixed-age sample, but no analyses examining age or menopausal status [3] Sample was mixed in both gender and age, but no gender-by-age interaction analysis [4] Sample all male or all under age 50 [5] Insufficient details to interpret results for older women [6]
Peer-reviewed	Not peer-reviewed [7]
Either primary research or meta-analyses	Secondary research literature (e.g., review papers, commentaries, editorials) [8]
Alcohol-relevant outcomes	Primary outcomes not alcohol-related, such as only mentioning or peripherally examining alcohol use (e.g., as one of several variables in multivariate analysis) [9]
Provides clinically relevant results, beyond epidemiology/prevalence of alcohol use or overall mortality	Focused on numbers, prevalence, and epidemiology of problem alcohol use among female populations (articles on mortality due to specified alcohol-related diseases were included) [10, 11]
Findings conveyed clinical implications	Focused on psychometrics, association of alcohol use with non-alcohol health service utilization, or public policy outcomes [12–14] Examined the effects of alcohol on nonclinical or nonspecific mechanisms (e.g., while heart rate variability is a relevant mechanism for many health conditions, articles that did not examine such alcohol and heart rate variability in relation to a clinical outcome were excluded) [15] Focused on clinical outcomes that were not in the review’s scope or very limited number of articles on a given topic (e.g., one article on alcohol and glaucoma) [16]
Results generalizable across historical and situational context	Focused on event-specific outcomes; (e.g., articles related to the COVID-19 pandemic or natural disasters) [17]
Sample includes women with heavy drinking, binge drinking, and/or AUD	Controlled feeding/alcohol administration studies that excluded women with current or past heavy alcohol use, AUD, or a positive family history of AUD [18]
***Additional Criteria Used During Critical Appraisal Phase***	
NA	Problematic distribution of alcohol use among female participants (e.g., very low alcohol use in female participants or few female participants who drink) [19]
NA	Nonstandardized or unreliable methods used to assess alcohol use or AUD [20]
NA	Sampling strategy and/or sample characteristics significantly limited generalizability of the findings [21]
NA	Insufficient information regarding alcohol use assessment and/or other critical information (e.g., number of female participants in sample) [22]
NA	Methodological (not alcohol-related) issues [23]

*Note:* NA, not applicable.

**Appendix 1:** Extraction Table of Reviewed Articles Related to Correlates of Drinking

Author	Study Method	Quitters a	Sex Differences^b^	Age Differences c	Country	Total *n*, % (Female)	Total *n*, % (Male)	Participant Age (Years)‡	Summary of Findings
***Biological Correlates***									
Laberge (2021)^57^	Cross-sectional study of associations between health and heavy drinking in older adults	N	N	N	Canada	1,324 (58%)	950 (42%)	Range*: 65+ M(SD) = 73(6)	Compared to women who did not drink, those who consumed ≥ 1 drink/week were less likely to self-rate physical health as poor. Women who did not drink were more likely than those who did to be ill or disabled before retirement. Wo men consuming ≥ 7 drinks/week were more likely to be living with a spouse or partner, compared to those who did not drink or those who consumed 1–2 drinks/week.
Al-Rousan (2022)^58^	Cross-sectional correlates with binge drinking in a sample age 65+	N	N	N	USA	10,328 (55%)	8,466 (45%)	Range: 65+ M = 76	Compared to women who did not binge drink in the past month, those who did binge drink had a lower prevalence of chronic disease, higher education, and higher prevalence of tobacco or cannabis use.
Satre (2007)^59^	Cross-sectional study on physical health correlates with various patterns of alcohol use	Y	N	N	USA	3,346 (50%)	3,316 (50%)	Range: 65–90	Presence of medical conditions (e.g., heart problems, arthritis, anxiety) and worse self-rated health were higher among women who did not drink in the past 12 months (but had previously been drinking) compared to those who drank < 7 drinks/week. Among those with past-year drinking, women who drank ≥ 7 drinks/week reported fewer heart/cholesterol problems but had higher likelihood of unhealthy behaviors (cigarette use, sedentary lifestyle) compared to those who drank < 7 drinks/week.
Balsa (2008)^60^	Cross sectional associations of alcohol and health among older adults	Y	N	N	USA	2,587 (38%)	4,274 (62%)	Range: 65+ M = 76	Any alcohol consumption up to 2 drinks/day by women was associated with better self-perceived health status and with lower use of health services; this association of drinking and health was diminished with increasing alcohol use.
Stelander (2022)^61^	Cross-sectional study examining correlates of at-risk drinking per AUDIT	Y	N	N	Nordic	4,451 (52%)	4,165 (48%)	Range: 60+ 60% 60–69 31% 70–79 9% 80+	Among women, being at risk of AUD (AUDIT-C score ≥ 3) and problem drinking (per AUDIT individual items) was associated with lower age and higher education. Better self-reported health, living with a spouse/partner, and more social support were associated with greater likelihood of at-risk drinking, but lower likelihood of reporting problems/consequences related to drinking. Mental distress and the use of sleeping pills were associated with higher likelihood of at least one alcohol-related problem.
Brennan (2005)^62^	Three-year longitudinal survey on pain and alcohol-related problems or consequences	Y	Y	N	USA	154 (38%)	247 (62%)	Range*: 55–65 at baseline M* = 68 at follow-up	At baseline, those who reported ≥ 1 alcohol-related problems on the DPI (versus zero) were more likely to use alcohol for pain management. High frequency of alcohol use to manage pain at baseline predicted more severe drinking problems at 3-year follow-up. Sex differences were tested, with findings stronger among men; authors attributed this to the higher rates of alcohol-related consequences reported by men than women in this general population sample.
Stevenson (2005)^63^	Cross-sectional study on predictors of alcohol use among older women	N	N	N	USA	135 (100%)	0 (0%)	Range: 60+ M = 69	“Best predictor model” of alcohol use included regular use of > 1 over-the-counter medications, heavier coffee consumption, and use of alcohol to fall asleep.
***Psychological Correlates—Distress, Mood, and Stress***									
Behrendt (2021)^72^	Latent class analysis of older men/women who consumed 12/6 U.S. standard drinks per week	Y	Y	Y	Nordic	7,194 (51%)	7,041 (49%)	M(SD)* ages 55–64 = 60(3) ages 65–74 = 69(3)	Compared to men, women ages 55–64 were more likely to report distress due to pain, sleep, and tiredness, and those ages 65–74 were more likely to report distress and impairment related to pain and physical health.
Choi (2011)^73^	Secondary analysis of U.S. national survey, consisting of self-reports and interviews	N	S	N	USA	2,863 (54%)	2,462 (46%)	Range: 50 + 57% 50–64 43% 65+	Compared to women who did not use alcohol during past month, women who consumed ≥ 3 drinks on one occasion in the past month had higher psychological distress, an effect not found in men. Compared to non-binge-drinking and abstinent women, binge drinking (in these analyses, defined as ≥ 5 drinks) women had higher psychological distress, fewer medical conditions (but not different self-rated overall health), initiated alcohol use at a younger age, and were more likely to have received alcohol treatment. Women who binge drank reported more social support and resources than those who abstained and those who did not binge drink.
Choi (2011)^74^	Secondary analysis of U.S. national survey, consisting of self-reports and interviews	N	S	N	USA	2,826 (54%)	2,436 (46%)	Range: 50+	After controlling for sociodemographic and other variables, there was no relationship between consuming up to 2 drinks per day and psychological distress among women.
Sacco (2014)^75^	Cross-sectional study of national survey data on stress and drinking	N	Y	N	USA	2,231 (51%)	2,129 (49%)	Range: 60+ M = 71	In men and women, more stressful life events were associated with increased odds of past-year AUD, but not heavy drinking or drinking quantity/frequency in the past year. Higher perceived stress was associated with risk of AUD for men but not for women. Among women but not men, never being married (compared to divorced/widowed/separated) was associated with lower risk of past-year AUD.
Choi (2017)^76^	Cross-sectional national survey	N	S	Y	USA	7,811 (53%)	6,927 (47%)	Range: 50+ M = 64	Adverse childhood experiences (e.g., physical/sexual abuse, parental substance abuse) were associated with increased odds of AUD in the combined sample of men and women over age 50. In this general population sample that did not account for lifetime history of AUD, associations of certain types of adverse childhood experiences were more strongly associated with AUD in men than in women; however, analyses were stratified.
Rudolph (2013)^77^	Cross-sectional study	Y	S	N	USA	749 (66%)	391 (34%)	Range: 50+	Among nonabstinent participants (in the past month), adverse neighborhood conditions that evoked stress or fear were associated with more binge drinking for female but not male participants (in stratified analyses).
Jemberie (2020)^78^	Latent class analysis of participant responses on the Addiction Severity Index	Y	Y	N	Nordic	1,255 (72%)	492 (28%)	Range: 50+ M* = 58	Among individuals who reported being “troubled by an alcohol problem” in the past month, the highest proportion of women (47%) belonged to Class 3 (late onset alcohol problem, co-occurring anxiety/depression), with most of this class also endorsing histories of emotional and physical abuse; fewer men belonged to this class. Women were less likely than men to belong to classes characterized by early onset of alcohol problems.
***Psychological Correlates—Psychiatric Comorbidities***									
Jemberie (2020)^78^	Latent class analysis of participant responses on the Addiction Severity Index	Y	Y	N	Nordic	1,255 (72%)	492 (28%)	Range: 50+ M* = 58	Among individuals who reported being “troubled by an alcohol problem” in the past month, the highest proportion of women (47%) belonged to Class 3 (late onset alcohol problem, co-occurring anxiety/depression), with most of this class also endorsing histories of emotional and physical abuse; fewer men belonged to this class. Women were less likely than men to belong to classes characterized by early onset of alcohol problems.
Schiller (2023)^81^	Survey on drinking, loneliness, and self-reported depression in a random sample of adults in a Swedish population register	N	S	N	Nordic	4,988 (49%)	5,108 (51%)	Range: 70–84 47% 70–74 33% 75–79 20% 80–84	After adjusting for demographic and lifestyle factors (e.g., age, education, economic stress, physical activity, etc.), depression was more likely among women with AUDIT-C score ≥ 5 (suggesting “risky drinking”) compared to those with lower scores and those who did not drink in the past year. Among men, both those with risky drinking and those who did not drink in the past year had higher likelihood of depression than those with lower AUDIT-C scores.
Lin (2014)^82^	Cross-sectional analysis of national survey data	Y	S	N	USA	4,759 (58%)	3,446 (42%)	Range: 65+ 46% 75+	Women age 65+ with a lifetime mood disorder were more likely than those without a lifetime mood disorder to have a diagnosis of current or lifetime AUD. Men and women with current AUD were three times more likely to have current or lifetime tobacco use disorder than those without current AUD.
van Gils (2023)^83^	Cross-sectional study on the contribution of mental health, drinking motives, and resilience to alcohol use by sex	Y	S	N	Western Europe	721 (51%)	683 (49%)	Range: 65–97 M(SD)* = 73(6)	Among women age 65+ who drank alcohol, smoking, drinking to cope, mood enhancement motives, and general self-efficacy were associated with higher AUDIT scores. Among women with high-risk drinking (AUDIT score ≥ 3), of these constructs, only drinking to cope and depression were associated with higher AUDIT scores; for their male counterparts, smoking, anxiety, and mood enhancement motives were associated with AUDIT scores.
Chou (2011)^84^	Secondary analysis of prospective study data to examine binge drinking and DSM-IV diagnoses	Y	S	N	USA	7,981 (59%)	5,461 (41%)	Range: 50+ 44% 50–64 58% 65–79 67% 80+	Compared to women who were currently drinking but did not binge drink, women who binge drank in the past year were more likely to have DSM-IV alcohol abuse/dependence. Women who binge drank in the past year, but less than monthly, were more likely than those who did not binge drink to have panic disorder and post-traumatic stress disorder.
Satre (2011)^85^	Assessment for recent substance misuse of patients seeking outpatient care for depression	N	N	N	USA	97 (62%)	57 (38%)	Range: 60+ M(SD)* = 68(7)	Among women age 60+ who sought treatment for depression at an outpatient clinic, 27% reported having ≥ 5 drinks on at least one occasion in the prior year; 13% reported using cannabis, 18% sedatives, and 16% tobacco in the prior year.
Liang (2021)^86^	Pooled analysis of three multinational samples with follow up of 4–18 years	Y	N	N	USA; UK; East Asia	16,365 (56%)	13,141 (44%)	Range of M(SD): 57–65 (9–11)	Among participants without depression at baseline, women drinking ≤ 7 drinks/week had a lower incidence of depressive symptoms over time compared with those who never drank. Rates were not different between those who never drank, who had quit drinking, or were currently drinking heavily (> 7 drinks/week).
Carvalho (2018)^87^	2-year prospective study examining alcohol use and depression and anxiety symptoms, by sex	N	Y	N	UK	3,110 (52%)	2,870 (48%)	Range: 50+ M(SD)* = 63(9)	Problem drinking (versus nonproblem drinking), as determined by the CAGE screener, was associated with higher risk of later anxiety and depression for women to a greater extent than for men during the 2-year follow-up. The findings were not significant when tested with the overall mixed sex sample.
Keyes (2019)^88^	10-year longitudinal multinational study of alcohol use and depressive symptoms	Y	Y	N	Multiple	30,356 (53%)	26,920 (47%)	Range: 50+ M* = 64	The study compared five groups: those who were currently abstaining; those who had been abstinent for a long time (across the 10-year period); those who drank weekly but ≤ 2 drinks per day for women (≤ 3 drinks for men) and had no binge drinking; and those who drank weekly with > 2 drinks on drinking days for women (> 3 drinks for men) and/or binge drinking. Women who drank alcohol at any level had higher incidence of depression than any men in any drinking category.
Tait (2012)^89^	Parent study pooling nine longitudinal studies on alcohol, depression, and sex	N	S	N	Australia	31,202 (80%)	7,901 (20%)	Range* = 45–103 Median* = 60	Women who were abstinent or consumed more than ~1.5 drinks/day had higher likelihood of depression, compared with those who consumed less than ~1.5 drinks/day. Women who were abstinent at all study timepoints had increased odds of depression compared to those who drank < 3 drinks/day.
***Psychological Correlates—Clinical Presentation and Course***									
Jemberie (2020)^78^	Latent class analysis of participant responses on the Addiction Severity Index	Y	Y	N	Nordic	1,255 (72%)	492 (28%)	Range: 50+ M* = 58	Among individuals who reported being “troubled by an alcohol problem” in the past month, the highest proportion of women (47%) belonged to Class 3 (late onset alcohol problem, co-occurring anxiety/depression), with most of this class also endorsing histories of emotional and physical abuse; fewer men belonged to this class. Women were less likely than men to belong to classes characterized by early onset of alcohol problems.
Mejldal (2020)^91^	DSM-5 AUD typologies in relation to demographics and treatment outcomes	Y	Y	N	Nordic	122 (36%)	219 (64%)	Range: 60+ Median = 65	Three classes of AUD typology were defined among older men and women engaged in a clinical treatment trial for alcohol use. Women were more likely than men to belong to a class distinguished by symptoms of irresistible cravings, drinking more than intended, and continued use despite consequences.
Brennan (2010)^92^	Examination of predictors of changes in drinking over 10 years in men and women	Y	Y	Y	USA	529 (41%)	762 (59%)	Range: 55–65 M(SD)* = 61(3) at baseline	Alcohol consumption remained more stable over time for women compared to men. Sex did not predict changes in alcohol consumption over the 10-year follow-up period.
Molander (2010)^93^	Longitudinal study examining predictors of drinking changes in adults between ages 53 and 64	N	Y	N	USA	2,854 (54%)	2,429 (46%)	All participants age 53 at wave 1 and 64 at wave 2	For women, drinks per drinking day decreased while overall number of drinking days increased. Women were more likely than men to transition from drinking to becoming abstinent. Authors state that full examination of sex differences was outside of the scope of the paper.
Holdsworth (2017)^94^	Examination of baseline predictors and course of alcohol consumption among older adults over 10-year period	N	S	N	UK	2,635 (57%)	2,016 (43%)	Range: 50+ M = 62 at baseline	Higher income, more education, being employed, and smoking were associated with relatively more frequent drinking and more drinks on drinking days in women (measured as continuous variables). Women who had no partner or who had lost/separated from their partner (versus those with a romantic partner) had the steepest decline in drinking over 10 years. Deterioration in health over time was associated with decreases in the number of drinks per week compared to those with consistently poor health or improved health over time.
Dauber (2018)^95^	Analysis of data from adults in addiction treatment	Y	Y	N	Western Europe	3,771 (35%)	7,089 (65%)	Range: 60+ M = 65	Senior women had positive treatment outcomes. Compared to men, women were more likely to be widowed and living alone, differed in reasons for drinking, had higher retirement rates, and had later AUD onset. Frequent contacts with doctors could play an important role in detection of AUD and initiation of treatment.
Holton (2019)^96^	Longitudinal study on alcohol use, health, and retirement	N	S	N	Ireland	2,353 (55%)	1942 (45%)	Range: 50+ M = 62 at baseline	Women with self-reported poor versus good health drank less at baseline. Increased drinking frequency over time was associated with higher education and tobacco use in men and women. Binge drinking (defined as 4.3 U.S. standard drinks/occasion) was less frequent among rural versus urban women. Those who did not drink and had lower education and fair health were most likely to be lost to attrition, potentially skewing results.
Brennan (2011)^97^	Latent growth model of baseline predictors of alcohol use and alcohol-related problems over 20 years	Y	Y	Y	USA	320 (45%)	399 (56%)	Range: 55–65 M(SD)* = 61(3) at baseline	For men and women, social approval of alcohol use and reported use of substances for tension reduction correlated with more alcohol use and related consequences at baseline but were not associated with changes in drinking over 20-year follow-up for women. From baseline to 10-year follow-up, both men and women reported decreased alcohol-related problems, leveling off between 10- and 20-year follow-up. Baseline alcohol-related problems were associated with earlier decline in drinking among older women compared to men.
***Psychological Correlates—Co-Occurring Tobacco Use***									
Al-Rousan (2022)^58^	Cross-sectional correlates with binge drinking in a sample age 65+	N	N	N	USA	10,328 (55%)	8,466 (45%)	Range: 65+ M = 76	Compared to women who did not binge drink in the past month, those who did binge drink had a lower prevalence of chronic disease, higher education, and higher prevalence of tobacco or cannabis use.
Lin (2014)^82^	Cross-sectional analysis of national survey data	Y	S	N	USA	4,759 (58%)	3,446 (42%)	Range: 65+ 46% 75+	Women age 65+ with a lifetime mood disorder were more likely than those without a lifetime mood disorder to have a diagnosis of current or lifetime AUD. Men and women with current AUD were three times more likely to have current or lifetime tobacco use disorder than those without current AUD.
Holton (2019)^96^	Longitudinal study on alcohol use, health, and retirement	N	S	N	Ireland	2,353 (55%)	1942 (45%)	Range: 50+ M = 62 at baseline	Women with self-reported poor versus good health drank less at baseline. Increased drinking frequency over time was associated with higher education and tobacco use in men and women. Binge drinking (defined as 4.3 U.S. standard drinks/occasion) was less frequent among rural versus urban women. Those who did not drink and had lower education and fair health were most likely to be lost to attrition, potentially skewing results.
Blazer (2009)^99^	Secondary, cross-sectional data analysis of a national survey on alcohol use and binge drinking in adults age 50+	N	S	Y	USA	6,001 (55%)	4,952 (45%)	Range: 50+	For men and women, binge drinking (defined as ≥ 5 drinks) was associated with tobacco and illicit drug use compared to not drinking (not accounting for former drinking). Among women only, nonprescribed drug use was associated with binge drinking; marital status was associated with drinking patterns in men but not women. Results varied when binge drinking was compared to not drinking versus other patterns of alcohol use, for men and women.
Ahlner (2022)^100^	Cross-sectional study on the association of health and sociodemographic factors with alcohol consumption	Y	Y	N	Nordic	617 (53%)	539 (47%)	Range: 70	Lifetime alcohol abstention was associated with lower physical activity for women but not men. In women (not men), smoking tobacco was associated with 7–14 drinks per week but no other drinking categories (i.e., there was not a linear association). Compared to women consuming ≥ 7 drinks per week, former drinking women had higher education and lower childhood SES (associations not found in men).
***Social Correlates***									
Al-Rousan (2022)^58^	Cross-sectional correlates with binge drinking in a sample age 65+	N	N	N	USA	10,328 (55%)	8,466 (45%)	Range: 65+ M = 76	Compared to women who did not binge drink in the past month, those who did binge drink had a lower prevalence of chronic disease, higher education, and higher prevalence of tobacco or cannabis use.
Stelander (2022)^61^	Cross-sectional study examining correlates of at-risk drinking per AUDIT	Y	N	N	Nordic	4,451 (52%)	4,165 (48%)	Range: 60+ 60% 60–69 31% 70–79 9% 80+	Among women being at risk of AUD (AUDIT-C score ≥ 3) and problem drinking (per AUDIT individual items) were associated with lower age and higher education. Better self-reported health, living with a spouse/partner, and more social support were associated with greater likelihood of at-risk drinking, but lower likelihood of reporting problems/consequences related to drinking. Mental distress and the use of sleeping pills were associated with higher likelihood of at least one alcohol-related problem.
Holdsworth (2017)^94^	Examination of baseline predictors and course of alcohol consumption among older adults over 10-year period	N	S	N	UK	2,635 (57%)	2,016 (43%)	Range: 50+ M = 62 at baseline	Higher income, more education, being employed, and smoking were associated with relatively more frequent drinking and more drinks on drinking days in women (measured as continuous variables). Women who had no partner or who had lost/separated from their partner (versus those with a romantic partner) had the steepest decline in drinking over 10 years. Deterioration in health over time was associated with decreases in the number of drinks per week compared to those with consistently poor health or improved health over time.
Dauber (2018)^95^	Analysis of data from adults in addiction treatment	Y	Y	N	Western Europe	3,771 (35%)	7,089 (65%)	Range: 60+ M = 65	Senior women had positive treatment outcomes. Compared to men, women were more likely to be widowed and living alone, differed in reasons for drinking, had higher retirement rates, and had later AUD onset. Frequent contacts with doctors could play an important role in detection of AUD and initiation of treatment.
Holton (2019)^96^	Longitudinal study on alcohol use, health, and retirement	N	S	N	Ireland	2,353 (55%)	1942 (45%)	Range: 50+ M = 62 at baseline	Women with self-reported poor versus good health drank less at baseline. Increased drinking frequency over time was associated with higher education and tobacco use in men and women. Binge drinking (defined as 4.3 U.S. standard drinks/occasion) was less frequent among rural versus urban women. Those who did not drink and had lower education and fair health were most likely to be lost to attrition, potentially skewing results.
Blazer (2009)^99^	Secondary, cross-sectional data analysis of a national survey on alcohol use and binge drinking in adults age 50+	N	S	Y	USA	6,001 (55%)	4,952 (45%)	Range: 50+	For men and women, binge drinking (defined as ≥ 5 drinks) was associated with tobacco and illicit drug use compared to not drinking (not accounting for former drinking). Among women only, nonprescribed drug use was associated with binge drinking; marital status was associated with drinking patterns in men but not women. Results varied when binge drinking was compared to not drinking versus other patterns of alcohol use, for men and women.
Ahlner (2022)^100^	Cross-sectional study on the association of health and sociodemographic factors with alcohol consumption	Y	Y	N	Nordic	617 (53%)	539 (47%)	Range: 70	Lifetime alcohol abstention was associated with lower physical activity for women but not men. In women (not men), smoking tobacco was associated with 7–14 drinks per week but no other drinking categories (i.e., there was not a linear association). Compared to women consuming ≥ 7 drinks per week, former drinking women had higher education and lower childhood SES (associations not found in men).
Bosque-Prous (2017)^101^	Cross-sectional study on individual and environmental correlates of hazardous drinking (defined by positive AUDIT-C screen)	N	S	Y	Multiple	35,892 (54%)	30,063 (46%)	Range: 50+ 49% 50–64 51% 65+	Screening positive on the AUDIT-C (≥ 4 for women, ≥ 5 for men) was associated with tobacco use among men and women. Women who perceived their health as poor to fair (compared to good to excellent) and who were divorced/widowed (compared to living with a spouse) were less likely to drink hazardously (for the latter, the opposite trend was found for men). Women, but not men, with upper secondary education (compared to less education) were more likely to screen positive on AUDIT-C.
Reczek (2016)^102^	Mixed-methods study examining marital status and alcohol use over 18 years	N	Y	Y	USA	6,058 (60%)	4,003 (40%)	M(SD) = 61(9) at baseline	Being married, including remarried, increased likelihood of women engaging in heavy drinking (≥ 3 drinks at least 1 day/week in past 3 months) versus being never married or previously married. The opposite was found for men. Divorced women who drank decreased their drinking more quickly as they aged, compared with stably married women who drank.
Villalonga- Olives (2020)^103^	Longitudinal study examining changes in social capital in relation to binge drinking	N	S	N	USA	3,931 (54%) at wave 3	2,719 (46%) at wave 3	Range: 50+ M(SD)* = 67(10) at baseline	Higher education was associated with higher probability of no binge drinking in later years among men and women. Positive social support and neighborhood social cohesion were protective factors for binge drinking to a greater extent for older women than men.
Bryan (2017)^104^	Survey about heavy drinking in lesbian, gay, and bisexual women and men	N	S	Y	USA	1,081 (46%)	1,270 (54%)	Range*: 50–98 M(SD)* = 61(8)	Among lesbian and bisexual older women, current smoking, younger age, income > 200% of the federal poverty level, not being in recovery from alcohol or drugs, greater social support, and lower perceived stress were all associated with increased likelihood of heavy drinking compared to no drinking.
Iparraguirre (2015)^105^	Longitudinal study of changes in alcohol use over time	N	S	N	UK	Not provided	Not provided	Range: 50+ M* = 67 at wave 5	Women, but not men, were more likely to report consuming ≥ 3 drinks per week if they were relatively younger and had higher income, and less likely to drink ≥ 3 drinks/week if they identified as a caregiver. For men and women, consuming ≥ 3 drinks/week was associated with higher education and more cigarette use.
León-Muñoz (2015)^106^	Cross-sectional study using phone interviews and home visits	Y	Y	Y	Western Europe	1,668 (55%)	1,390 (45%)	Range: 60+	Alcohol consumption was lower among women age 75+ compared to women ages 60–74. In women but not men, higher education (completed secondary education or university) was associated with drinking < 1.7 drinks/day, but not more than that amount.
Towers (2018)^108^	Secondary data analysis of a longitudinal survey that looked at correlates of alcohol use	Y	S	Y	New Zealand	1,529 (52%)	1,399 (48%)	Range: 52–86 M(SD) = 66(8)	For both men and women over age 50, the association between drinks per day and general physical health (self-reported functional health and well-being) followed the same trends as the association between drinks per day and SES, suggesting confounding covariance between SES and alcohol use in relation to health. The association of drinking and health was substantially reduced after controlling for a direct measure of SES.

‡ Participants’ age range and mean (standard deviation), if provided. Values for female participants only are provided when available.

* Statistics provided only for the combined male and female sample.

a **Quitters:** “Y” indicates that the study accounted for participants who had a history of heavy alcohol use or AUD, accounting for possible “sick quitter” effects in their analyses. Articles either (1) assessed for past drinking habits or symptoms of AUD and then accounted for this data in their statistical analyses or (2) limited their sample to current alcohol users only. “N” indicates that the study did not assess for past alcohol use/AUD or account for it in analyses.

b **Sex Differences:** “Y” indicates that male and female participants were directly compared in analyses. “N” indicates that they were not compared. “S” indicates studies that ran stratified or otherwise separated analyses for male and female participants.

c **Age Differences:** “Y” indicates that age was tested in relation to outcomes, either as a continuous or categorical (e.g., comparing 60–69 to 70–79, etc.) variable; “N” indicates that the full sample was ≥ 50 years old and no age analyses were conducted.

*Note:* AUD, alcohol use disorder; AUDIT, Alcohol Use Disorders Identification Test; AUDIT-C, Alcohol Use Disorders Identification Test–Concise; DPI, Drinking Problems Index; SES, socioeconomic status.

**Appendix 2:** Extraction Table of Reviewed Articles Related to Physical as Well as Cognitive and Neuropsychological Consequences of Alcohol Use in Older Women

Author	Study Method	Quitters a	Sex Differences^b^	Age Differences c	Country	Total *n*, % (Female)	Total *n*, % (Male)	Participant Age (Years)‡	Summary of Findings
***Breast Cancer***									
Tin Tin (2024)^49^	Meta-analysis of 14 prospective cohort studies on the effects of alcohol on sex hormones; examined alcohol dehydrogenase (*ADH1B*) gene in relation to drinking and hormones	NK	NA	Y	USA; UK; Western Europe; Nordic; Netherlands	218,907 (100%)	0 (0%)	Range: 43–45 (premenopausal) 60–63 (postmenopausal) women	Higher alcohol intake was associated with higher estradiol levels in post-, but not premenopausal women, and with higher levels of several other sex hormones in both pre- and postmenopausal women. Higher alcohol intake was associated with lower sex-hormone binding globulin (SHBG) in postmenopausal women. There was evidence of a potential shared causal locus at the *ADH1B* gene for alcohol intake, higher free testosterone, and lower SHBG.
Key (2006)^116^	Meta-analysis of primary breast cancer and alcohol use	NK	NA	N	Multiple	75,728 (100%)	0 (0%)	Not provided	Any alcohol use was associated with 22% excess risk of breast cancer, compared to women who did not drink. Each 10g of ethanol/day (0.7 drinks) was associated with added 10% risk of breast cancer, unrelated to menopausal status.
Rustagi (2021)^117^	Case-control study on alcohol use and breast cancer risk	N	NA	N	USA	5,898 (100%)	0 (0%)	M(SD) = 57(12)	Postmenopausal women who consumed ≥ 1 drink/day had increased risk versus those who did not drink. Absolute breast dense volume mediated 25% of the association between drinks and breast cancer risk.
Tjønneland (2006)^118^	Prospective study on alcohol use, folate intake, and breast cancer risk	N	NA	N	Nordic	776 (100%)	0 (0%)	Range: 50–64	Alcohol intake was associated with higher risk of breast cancer and was strongest among women with low folate intake; folate intake may buffer alcohol’s effect on risk of breast cancer among postmenopausal women.
McCarty (2012)^119^	Nested case-control study on alcohol use and genotype in relation to breast cancer risk	N	NA	N	USA	2,111 (100%)	0 (0%)	M(SD) = 63(5)	Any drinking increased the risk of breast cancer, and drinking ≥ 3 drinks per day was associated with the greatest risk compared to other consumption levels. The *ADH1B* genotype interacted with alcohol intake to increase risk of breast cancer.
Hahn (2018)^120^	Case-cohort study on interactive effects of alcohol intake and genotypes in the alcohol metabolism pathway	Y	NA	N	Netherlands	2,931 (100%)	0 (0%)	Range: 55–69 M(SD) = 61(4)	Over 20 years, there was an increase in breast cancer risk for women consuming more than approximately 2 drinks per day (compared to those who abstained) and for every ~0.7 drinks/day increase in alcohol intake. There was no interaction between alcohol and genotype for risk of breast cancer. Sensitivity analyses were run to account for former drinkers; authors suggest that there was some difference in results but did not elaborate.
Park (2009)^121^	Analysis of alcohol use at baseline and followup for 7 years using state cancer registries	N	NA	N	USA	184,418 (100%)	0 (0%)	Range: 50–71	Consuming 0.7 drinks/day increased breast cancer risk, with increasing risk up to ≥ 2.5 drinks/day (the highest level assessed). Alcohol intake was positively associated with estrogen receptor (ER)-positive, progesterone receptor (PR)-positive tumors, and ER+/PR+ tumors, but not with ER−, PR−, or ER−/PR− tumors. The association was stronger in women using hormone therapy for 10+ years.
Li (2010)^122^	Secondary analysis of a prospective cohort study on alcohol and breast cancer subtypes	Y	NA	N	USA	87,724 (100%)	0 (0%)	Range: 50–79	Among women who currently drank, number of drinks per week was positively associated with risk of invasive breast cancers, particularly with invasive lobular carcinoma and hormone receptor-positive tumors. Compared with those who had never consumed alcohol, those who consumed ≥ 14 drinks per week had a 2.13-fold increased risk of lobular, but not ductal, carcinoma.
Sun (2020)^123^	Meta-analysis of prospective cohort studies on alcohol use and breast cancer risk	NK	NA	N	Multiple	Not provided	0 (0%)	Not provided	A linear trend for breast cancer risk by alcohol dose existed up to approximately 4 drinks/day. For postmenopausal women, risk increased by 11% for every 0.7 drinks/day (10 g/day of total alcohol) and increased by 23% for every ~1.5 drinks/day. The largest effects were found for hormone positive subtypes.
Hvidtfeldt (2015)^124^	Secondary analysis of two prospective cohort studies on interactive effects of alcohol and hormone therapy on breast cancer risk	N	NA	N	Nordic	30,789 (100%)	0 (0%)	Range: 51–63 Median = 56	Among women not using hormone therapy, more alcohol use days/week was associated with higher breast cancer risk. Among women who used hormone therapy, more breast cancer cases (primarily ER+ subtype) were observed in those who consumed ≥ 7 drinks/week versus those who abstained. Estradiol and testosterone were elevated in women who used hormone therapy and consumed ≥ 7 drinks/week, compared to women who were abstinent and did not use hormone therapy.
Falk (2014)^125^	Secondary analysis of randomized trial with up to 13 years of followup	Y	NA	N	USA	54,562 (100%)	0 (0%)	Range: 55–74	Women consuming ≥ 7 drinks/week had 1.4 times the risk of any cancer compared to those who never drank. Women who had previously been drinking had a nonsignificant 16% higher risk of any cancer compared with those who never drank. Risk for mixed ductal/lobular tumors was higher with ≥ 7 drinks/week compared to fewer drinks per week, but samples were small for these analyses. PR status explained the positive association with ER status.
Nielsen (2008)^126^	Prospective study on alcohol use, hormone use, and breast cancer risk	N	NA	N	Nordic	5,035 (100%)	0 (0%)	Range: 39–91 M(SD) = 62(8)	Consumption of > 7 drinks/week was associated with increased risk of breast cancer, versus < 1 drink/week. Risk increased for every additional drink consumed per day. Women with hormone use who consumed > 14 drinks per week had a higher risk of breast cancer versus those who did not drink and did not use hormones.
Suzuki (2008)^128^	Meta-analysis of studies on alcohol intake and breast cancer subtypes; examined hormone status subtypes	NK	NA	Y	USA; Canada; Nordic; Australia; East Asia	Not provided	0 (0%)	Not provided	An increase in alcohol consumption of 0.7 drinks/day was associated with a 12% increase in risk of all ER+ tumors, 11% increase in risk of ER+PR+ tumors, and 15% increase in risk of ER+PR− tumors. Increase in risk was smaller (7%) for all ER− tumors. The relationship between alcohol intake and ER+ tumors was confined to studies in the United States, Canada, and Europe, but was not found in Asia or Australia. Postmenopausal hormone use, body mass index (BMI), and family history of breast cancer were relevant factors but did not fully explain alcohol results. Nine studies did not stratify based on menopausal status.
Platek (2009)^129^	Matched case study on lifetime alcohol use in relation to genetic markers of one-carbon metabolism	N	NA	Y	USA	2,953 (100%)	0 (0%)	M(SD) = 63(9)	Among postmenopausal (but not premenopausal) women, polymorphisms of the methylenetetrahydrofolate reductase (MTHFR) enzyme (critical to one-carbon metabolism, a process in cancer cell proliferation) were associated with increased breast cancer risk for women with above-the-median lifetime alcohol use (> 1.9 drinks/day) versus women who did not drink and had other genotypes.
Jung (2021)^130^	Genome-wide association gene-environment interaction study using data from a cohort study	N	NA	N	USA	10,179 (100%)	0 (0%)	Range: 50–79 M(SD) = 66(7)	Two C-Reactive Protein (CRP, an inflammatory biomarker) genotypes and lifestyle factors (alcohol intake, estrogen, and obesity) were predictors of breast cancer. Whether postmenopausal women consumed > ~1.3 drinks/day or fewer interacted with CRP genotype and lifestyle risk factors to predict cancer risk.
***Other Cancers***									
Floud (2023)^131^	Prospective cohort study on alcohol consumption and 21 types of cancer among post-menopausal women	Y	NA	N	UK	795,121 (100%)	0 (0%)	M(SD) = 56(5)	With mean follow-up of 17 years, aerodigestive cancers were associated with alcohol intake, and smoking interacted with drinking to increase risk. Every ~0.5 drinks/day increase in alcohol intake was associated with an increased risk of esophageal squamous cell carcinoma and cancers of the oral cavity, pharynx, larynx, breast, colon/rectum, pancreas, and lung; there was no change in risk or a decreased risk for some other cancers.
Razzak (2011)^132^	Prospective cohort study on alcohol and colorectal cancer	N	NA	N	USA	38,001 (100%)	0 (0%)	Range: 55–69 M(SD) = 62(4)	There was no association between alcohol consumption and incidence of colorectal cancer. Alcohol intake was not associated with colorectal cancer when analyzed by anatomic subsite or molecularly defined subtypes.
Kubo (2014)^133^	Secondary analysis of cohort data on drinking and skin cancer among postmenopausal women	Y	NA	N	USA	59,575 (100%)	0 (0%)	Range: 50–79 M = 64	Over a mean follow-up of 10.2 years, women consuming ≥ 7 drinks per week had greater risk of melanoma, and women consuming ≥ 1 drink per week had greater risk of nonmelanoma skin cancer, compared with women who did not drink. Every 7 additional drinks/week increased risk for both cancers.
Setiawan (2008)^134^	Secondary analysis of prospective cohort study on alcohol use and endometrial cancer risk	N	NA	N	USA	41,574 (100%)	0 (0%)	M = 61	Compared to women who did not drink, women consuming > ~1.7 standard drinks per day had a greater risk of endometrial cancer during average follow-up of 8.3 years.
Yang (2011)^135^	Secondary analysis of prospective cohort study on alcohol use and endometrial cancer risk	N	NA	Y	USA	114,414 (100%)	0 (0%)	Range: 50–71 M(SD) = 62(6)	There was no association between alcohol intake and endometrial cancer risk when women drinking 0–0.85, 0.85–1.7, and ≥ 1.7 standard drinks per day were compared to women who did not drink, regardless of beverage type.
***Cardiovascular Disease***									
Kadlecovà (2015)^137^	Cohort longitudinal study on alcohol consumption and stroke risk among men and women	NK	S	N	Nordic	6,404 (55%)	5,240 (45%)	Range*: ≤ 60 M(SD)* = 51(5) at baseline	During 43 years of follow-up, women who drank > 1.7 drinks/day versus those who drank less (> 0 but < 0.4 drinks/day) had higher stroke risk. Only 3% of the mixed-sex sample drank > 1.7 drinks per day, and article does not provide percentage for women. Unclear how former drinkers were accounted or in analyses.
Cunningham (2018)^138^	Cohort longitudinal study on current and past alcohol use at baseline and risk of stroke	Y	Y	N	USA	15,160 (56%)	12,105 (44%)	Range*: 45+ M(SD)* = 65(9)	During approximately 7 years follow-up, there was no stroke risk difference among women with different drinking habits. Among women specifically, any past regular drinking was associated with higher risk of stroke versus current drinking. Only 4% of women were in the “heavy drinking” group (≥ 7.5 drinks/week).
Conen (2008)^139^	Prospective cohort study on alcohol use and atrial fibrillation	N	NA	Y	USA	34,715 (100%)	0 (0%)	Median = 53–54	Women consuming ≥ 2 drinks/day had a 60% increased risk of incident atrial fibrillation versus women who did not drink.
Sahle (2018)^140^	Cohort longitudinal study on alcohol use and heart failure among women with hypertension	Y	S	N	Australia	3,093 (51%)	2,972 (49%)	Range*: 65–84 M(SD) = 72(5)	During a median follow-up of 11 years, there was no difference in risk of heart failure between women who never drank; those who previously drank; and those who consumed 1–7 drinks, 8–14 drinks, or > 14 drinks per week. Low incidence of heart failure in the sample may have limited statistical power.
Zureik (2004)^141^	Cohort longitudinal study on alcohol consumption and vascular structure among men and women	Y	Y	N	Western Europe	3,780 (61%)	2,436 (39%)	Range*: 65+ M* = 74	In sex-stratified analyses, among women age 65+, alcohol use was positively associated with increased diameter of common carotid arteries (CCA) but not associated with thickness of CCA or odds of atherosclerotic carotid plaque buildup. Only ~2% of the female sample drank > 1.7–2.5 drinks/day, and ~2% of the female sample drank > 2.5 drinks/per day.
***Liver Disease***									
Trembling (2017)^142^	Prospective cohort study of chronic liver disease in association to BMI and alcohol use	N	NA	N	UK	95,126 (100%)	0 (0%)	Range: 50–74 Median = 60	Among postmenopausal women ages 50–74, liver-related events were lowest among women drinking ≤ ~8.5 weekly drinks compared to women who abstained from alcohol and those drinking > ~8.5 drinks/week (no differentiation of lifetime abstinence v. former drinking, no timeframe for reported alcohol use provided); the same pattern was observed across all BMI categories.
Loomba (2009)^143^	Association of alcohol use and BMI with serum markers of liver disease	N	S	N	USA	1,277 (54%)	1,087 (46%)	M(SD)* = 70(11)	Among women (and men), there was no association between alcohol use and risk of liver injury when comparing those consuming < 3 drinks/day to those who did not drink (no distinction of lifetime abstinence versus former drinkers); there was an increase in markers of liver disease among those drinking > 3 drinks/day.
***Musculoskeletal Conditions***									
Kwon (2017)^144^	Cross-sectional examination of alcohol and sarcopenia	N	N	N	East Asia	2,373 (100%)	0 (0%)	M = 62	Among postmenopausal women, an AUDIT score ≥ 15 was associated with a higher risk of sarcopenia (muscle loss) compared to lower AUDIT scores.
Jang (2017)^145^	Cross-sectional examination of alcohol use and bone density	N	N	N	East Asia	3,312 (100%)	0 (0%)	M = 63	Bone mass density (BMD) was greater for women who drank at least monthly and less than twice per week than for those who did not drink and those who drank more than twice per week. Those who drank alcohol more than four times per week had lower BMD than all other groups. Those who did not drink and those who drank on more than 2 days per week had higher risk of osteoporosis than the group that drank at least monthly, but less than twice weekly. The analysis did not account for past drinking.
Paccou (2015)^146^	Associations of bone micro-architecture measures and alcohol use	N	S	N	UK	178 (47%)	198 (53%)	Range*: 72–81 M(SD) = 76(3)	Alcohol use was associated with both detrimental and beneficial effects on different measures of bone health among women. Findings were limited by the small number of women (n = 23) in the highest alcohol consumption group.
Marrone (2012)^147^	Intervention trial of alcohol effects on biochemical markers of bone health	Y	N	N	USA	40 (100%)	0 (0%)	M = 56(1)	Over a 14-day trial with women who were currently drinking, temporary abstinence was associated with markers of bone turnover for those who regularly drank ~1.4 drinks/day; drinking resumption was associated with reduced bone turnover.
***Other Health Outcomes***									
Kunzmann (2018)^148^	Cohort longitudinal study of mortality	Y	S	N	USA	51,306 (69%)	48,348 (31%)	Range*: 55–74 M(SD) = 65(10)	With a median follow-up of 9 years, there was a nonlinear association of drinking frequency and drinking intensity with overall mortality and cancer risk. Those had previously been drinking and those who were currently drinking heavily (> 2 drinks/day) were at increased risk of death compared to those who never drank.
Dam (2016)^149^	5-year longitudinal study on alcohol intake and health conditions among postmenopausal women	N	N	N	Nordic	21,523 (100%)	0 (0%)	Range: 54–73 M = 62	Women who increased alcohol intake from ≥ 14 drinks/week over a 5-year period had a higher mortality risk than women with a stable intake of 7–20 drinks/week. For these results, sensitivity analyses (e.g., excluding women who abstained, accounting for those who had previously been drinking) were not reported.
Herttua (2011)^150^	Longitudinal study on living arrangements and alcohol-related death (from national registers)	NA	S	Y	Nordic	3,279 (18%)	14,967 (82%)	Range*: 15–79	Between 2000 and 2007, the crude rate of alcohol-related mortality was higher for women living alone, compared to married or cohabitating women, for all age groups (ages 50–59, 60–69, and 70–79).
Yuan (2023)^151^	Analysis of epidemiologic trends in alcohol-associated fall injuries	Y	S	Y	USA	9,817,000 (35%)	18,482,259 (65%)	Range*: 65+	For women age 65+, rates of emergency department visits for alcohol-associated falls increased from 2011 to 2019 for all age subgroups (65–69, 70–74, 75–79), except for those age 80+.
Finkelstein (2007)^152^	Analysis of Medicare claimant data	N	S	N	USA	361,214 (60%)	240,708 (40%)	Range*: 65+ M* = 76	Women with an ICD-9 diagnosis of alcohol abuse had greater odds of a fall injury compared to women without alcohol abuse.
Sorock (2006)^153^	Case-control study analyzing association of alcohol use and fatal accidents	N	S	N	USA	9,088 (60%)	6,028 (40%)	Range*: 55+	Among women age 55+, those who were currently drinking had increased odds of fatal falls, fatal motor vehicle accidents, and death by suicide, compared to those who did not drink during follow-up. Drinking increased risk of suicide more for women than for men.
Sun (2011)^154^	Prospective cohort study of health-related behaviors predicting “successful aging”	N	N	N	USA	13,894 (100%)	0 (0%)	Median = 58	Among women age 70+, 1–2 drinks/day versus not drinking was associated with increased odds of “successful aging.” Pattern of alcohol use also predicted successful aging: Spreading out drinking at lower levels over the week was associated with less poor health than drinking higher quantities on fewer days.
Hu (2016)^155^	Longitudinal examination of drinking and physical functioning over 10 years	N	S	N	Western & Eastern Europe	15,255 (53%)	13,528 (47%)	Range: 45–69	Decline in physical function was not systematically associated with alcohol intake, problem drinking, or past drinking, but in this multi-national sample, country moderated findings. Among women who were currently drinking, physical function declined faster among Polish women consuming 1.4+ drinks/day compared to those drinking less and declined faster in Russian women consuming alcohol weekly compared to monthly (but not weekly). The analysis did not account for past drinking habits/patterns.
***Cognitive and Neuropsychological Consequences of Heavy Alcohol Use and AUD in Older Women***									
Nallapu (2023)^157^	Cross-sectional analysis of sex differences in alcohol use and cognition	N	S	N	Multiple	2,631 (59%)	1,801 (41%)	Range: 65–85 M(SD)* = 71(5)	Among women but not men who exhibited normal cognitive functioning, drinking 1–2 drinks per day was associated with better cognitive performance and lower Alzheimer’s disease risk compared to abstinence or drinking ≥ 3 drinks/day. The study did not differentiate or account for history of drinking.
Musich (2023)^158^	Cross-sectional survey study	N	Y	N	USA	124 (45%)	151 (55%)	Range: 50+ M(SD)* = 65(8)	Among women (but not men) with hypertension, a higher score on the AUDIT-C was associated with higher levels of subjective distractibility.
Tivis (2008)^159^	Cross-sectional study of the relationship among drinking, estrogen, and cognition	N	N	N	USA	298 (100%)	0 (0%)	Range of M(SD) across groups: 55–57 (4–5)	Alcohol use was not associated with episodic or semantic memory when controlling for confounding demographic, medical, and affective variables.
Hu (2023)^160^	Longitudinal study of AUD and dementia, and mediating role of cardiovascular disease	N	S	N	Nordic	140,069 (53%)	122,634 (47%)	Range: 40+ M(SD) = 59(13)	Women (and men) who were hospitalized for an alcohol-related health condition at age 40+ had higher risk of early onset (age < 65) and late-onset (age ≥ 65) dementia. The association between AUD and late-onset dementia was not mediated by cardiovascular disease (early onset mediational analyses were not sufficiently powered).
Bahorik (2021)^161^	Cohort study among older women Veterans	N	N	N	USA	4,414 (100%)	0 (0%)	Range: 55+ M = 65(6)	Female military Veterans age 55+ with ICD-9 alcohol-related diagnoses (including abuse or dependence) were three times more likely to have an ICD-9 diagnosis of dementia than those without alcohol-related diagnoses (and not in remission).
Zhang (2022)^162^	Retrospective cohort study examining the association of AUD with Alzheimer’s and Parkinson’s disease	N	S	N	USA	90,486 (35%)	167,878 (65%)	Range: 60 + M(SD) = 73(7)	Based on insurance claim records, women and men with ICD-9 or ICD-10 alcohol-related diagnoses had a higher risk of Alzheimer’s disease and, among white women only, a higher risk of Parkinson’s disease.
Liu (2019)^163^	Retrospective cohort study on alcohol consumption and dementia	N	S	N	East Asia	35,086 (66%)	18,225 (34%)	Range: 65+ M(SD)* = 71(8)	There was a lower risk of dementia in women who consumed ~3 drinks/day and no association among women consuming more than 3 drinks/day, compared with abstinent women (the study did not differentiate between women who never drank or had stopped drinking, and timeframes of alcohol assessment were not clearly described).
McGuire (2007)^164^	Longitudinal national survey of older adults using telephone interviews	N	S	N	USA	1,802 (66%)	914 (44%)	Range*: 70+ M* = 76	Among women age 70+, but not men, consuming < 1 drink/day was associated with lower odds of poor cognitive functioning compared with abstention (did not differentiate never/former drinking). The interview on cognitive functioning assessed mental status and memory.
Sabia (2014)^165^	10-year study on alcohol use and cognitive decline	N	S	N	USA	2,099 (29%)	5,054 (71%)	Range: 44–69 M = 56 at baseline	Across 10 years, women who did not drink for the 10-year period prior to baseline showed faster decline in measures of global cognitive function and executive function, compared to women drinking < 1 drink/day (only reference group used). Consuming ≥ 1.4 drinks per day was also associated with faster decline in executive function, however *p* = .09.
Corley (2011)^166^	Cross-sectional study on alcohol use and cognition	N	S	N	UK	474 (52%)	443 (48%)	M(SD)* = 70(1)	A small positive association between alcohol intake and memory/verbal ability was found among several cognitive assessments that were administered. Intelligence and socioeconomic status accounted for the association of alcohol and cognition.
Koch (2019)^167^	Cohort study on drinking and dementia in people with and without mild cognitive impairment	N	Y	N	USA	1,395 (46%)	1,626 (54%)	Range: 72+ Median* = 78	Among women age 72+ without mild cognitive impairment, consuming 1–7 drinks/week was associated with lower dementia risk than other drinking categories. Women who did not drink and those consuming > 14 drinks per week, compared to those who drank < 1 drink per week, had lower cognitive scores on the Modified Mini-Mental State Examination of global cognitive functioning.
Mewton (2023)^168^	Meta-analysis of 15 prospective epidemiological cohort studies	Y	S	N	Multiple	14,260 (58%)	10,218 (42%)	Range: 60–102 M(SD) = 72(8)	No association existed in women between alcohol use and dementia when controlling for education, BMI, depression, stroke, diabetes, myocardial infarction, hypertension, and high cholesterol and competing risk of death. There were no differences in findings between those who had abstained throughout life and those who had previously been drinking. Findings varied by continent of data collection.
Daviet (2022)^169^	Cross-sectional study on alcohol use and brain structure via imaging data	N	Y	N	UK	19,390 (53%)	17,288 (47%)	M(SD) = 63(7)	Among women and men drinking 0.7–1.5 drinks/day, higher alcohol intake was associated with lower global brain volume, regional gray matter volumes, and white matter microstructure, with increasing magnitude of effects as number of drinks increases.

‡ Participants’ age range and mean (standard deviation), if provided. Values for female participants only are provided when available.

* Statistics provided only for the combined male and female sample.

a **Quitters:** “Y” indicates that the study accounted for participants who had a history of heavy alcohol use or AUD, accounting for possible “sick quitter” effects in their analyses. Articles either (1) assessed for past drinking habits or symptoms of AUD and then accounted for this data in their statistical analyses or (2) limited their sample to current alcohol users only. “N” indicates that the study did not assess for past alcohol use/AUD or account for it in analyses. “NA”, used in treatment and prevention studies, reflects that accounting for sick quitters was not relevant because either they were all seeking treatment for AUD (in treatment studies) or did not necessarily need to have any alcohol-related problems to be part of the study (in prevention studies, if not related to the outcome measure, such as screening rates). “NK” indicates that whether the study accounted for sick quitters is not known.

b **Sex Differences:** “Y” indicates that male and female participants were directly compared in analyses. “N” indicates that they were not compared. “S” indicates studies that ran stratified or otherwise separated analyses for male and female participants. “NA” indicates that analysis of sex differences was not applicable because the study population only included women.

c **Age Differences:** “Y” indicates that age was tested in relation to outcomes, either as a continuous or categorical (e.g., comparing 60–69 to 70–79, etc.) variable; “N” indicates that the full sample was ≥ 50 years old and no age analyses were conducted.

*Note:* AUD, alcohol use disorder; AUDIT, Alcohol Use Disorders Identification Test; AUDIT-C, Alcohol Use Disorders Identification Test–Concise; BMI, body mass index; DSM, *Diagnostic and Statistical Manual of Mental Disorders*^29^; ICD, *International Classification of Diseases.*^28^

**Appendix 3:** Extraction Table of Reviewed Articles Related to Screening, Assessment, and Treatment Needs of Older Women With Heavy Drinking and Alcohol Use Disorder

Author	Study Method	Quitters a	Sex Differences^b^	Age Differences c	Country	Total *n*, % (Female)	Total *n*, % (Male)	Participant Age (Years)‡	Summary of Findings
***Screening and Assessment***									
Stevenson (2005)^63^	Cross-sectional study on predictors of alcohol use among older women	N	N	N	USA	135 (100%)	0 (0%)	Range: 60+ M = 69	“Best predictor model” of alcohol use included regular use of > 1 over-the-counter medications, heavier coffee consumption, and using alcohol to fall asleep.
Roberts (2005)^178^	Comparison of surgery patients in primary care who completed the AUDIT, SMAST-G, and alcohol use questions	N	N	N	UK	285 (57%)	215 (43%)	Range: 65+	The AUDIT performed better than the SMAST-G against U.K. Royal College of Psychiatrists’ guidelines on safe levels of alcohol consumption (which translate to < 8 U.S. standard drinks/week for women). The AUDIT performed best in older adult populations using sex-specific cut-offs for men (score of 5–6) and women (3–4).
Towers (2019)^179^	Comparison of the AUDIT-C with the older adult-targeted CARET risk evaluation tool	Y	N	N	New Zealand	1,795 (49%)	1,877 (51%)	Range: 50–89 M = 67	Among older women ages 50–89, 55% were classified as hazardous drinkers based on AUDIT-C scores, and 31% based on CARET scores. Only 1.4% of women were classified as having hazardous drinking on the CARET only. AUDIT-C scores of ≥ 4 for men and ≥ 3 for women were considered adequate thresholds for hazardous drinking for older adults.
Lemke (2005)^180^	Analysis of DSM-IV symptom sets and sequence of symptom onset for older men and women	Y	Y	N	USA	192 (28%)	486 (72%)	Range: 55–65 M = 69	Women first met DSM-IV alcohol criteria 5–6 years later than men, but in essentially the same order. The most common problems for women were tolerance and drinking more or longer than intended. Group-level symptom onset models, based on mixed sex and/or age samples, may not apply to older women.
Kuerbis (2013)^181^	Analysis of dimensionality and rank-order severity of DSM-IV alcohol abuse and dependence criteria among adults age 50+	Y	Y	N	USA	1,740 (51%)	1,672 (49%)	Range: 50+	Older women were less likely to endorse drinking larger amounts than intended, having legal problems, and interrupted role obligation than older men; however, the women who did endorse drinking larger amounts than intended did so at the lower end of the severity spectrum than men. Older men and women who are at risk for alcohol-related health problems are under identified. Diagnostic criteria are not sufficient to identify older adults’ risk for drinking problems.
Lewis (2018)^182^	Analysis of adherence to NIAAA guidelines and alcohol-associated risks in a community sample	Y	Y	Y	USA	292 (45%)	351 (55%)	Range: 21–70	Compared drinking patterns across age groups of adults. Single drinking measures (e.g., average consumption) may be inadequate metrics of risk, particularly in aging populations. Risks associated with exceeding guidelines are well substantiated but have not been su-ciently explored among older adults.
***Unique Treatment Needs—Triggers, High-Risk Situations, Relapse Antecedents, and Reasons for Using Alcohol***									
Al-Rousan (2015)^58^	Cross-sectional correlates with binge drinking in a sample age 65+	N	N	N	USA	10,328 (55%)	8,466 (45%)	Range: 65+ M = 76	Compared to women who did not binge drink in the past month, those who did binge drink had a lower prevalence of chronic disease, higher education, and higher prevalence of tobacco or cannabis use.
Stelander (2022)^61^	Cross-sectional study examining correlates of at-risk drinking per AUDIT	Y	N	N	Nordic	4,451 (52%)	4,165 (48%)	Range: 60+ 60% 60–69 31% 70–79 9% 80+	Among women being at risk of AUD (AUDIT-C score ≥ 3) and problem drinking (per AUDIT individual items) were associated with lower age and higher education. Better self-reported health, living with a spouse/partner, and more social support were associated with greater likelihood of at-risk drinking, but lower likelihood of reporting problems/consequences related to drinking. Mental distress and the use of sleeping pills were associated with higher likelihood of at least one alcohol-related problem.
Behrendt (2021)^72^	Latent class analysis of older men/women who consumed 12/6 U.S. standard drinks per week	Y	Y	Y	Nordic	7,194 (51%)	7,041 (49%)	M(SD)* ages 55–64 = 60(3) ages 65–74 = 69(3)	Compared to men, women ages 55–64 were more likely to report distress due to pain, sleep and tiredness, and those ages 65–74 were more likely to report distress and impairment related to pain and physical health.
Jemberie (2020)^78^	Latent class analysis of participant responses on the Addiction Severity Index	Y	Y	N	Nordic	1,255 (72%)	492 (28%)	Range: 50+ M* = 58	Among individuals who reported being “troubled by an alcohol problem” in the past month, the highest proportion of women (47%) belonged to Class 3 (late onset alcohol problem, co-occurring anxiety/depression), with most of this class also endorsing histories of emotional and physical abuse; fewer men belonged to this class. Women were less likely than men to belong to classes characterized by early onset of alcohol problems.
Lin (2014)^82^	Cross-sectional analysis of national survey data	Y	S	N	USA	4,759 (58%)	3,446 (42%)	Range: 65+ 46% 75+	Women age 65+ with a lifetime mood disorder were more likely than those without a lifetime mood disorder to have a diagnosis of current or lifetime AUD. Men and women with current AUD were three times more likely to have current or lifetime tobacco use disorder than those without current AUD.
Carvalho (2018)^87^	2-year prospective study examining alcohol use and depression and anxiety symptoms, by sex	N	Y	N	UK	3,110 (52%)	2,870 (48%)	Range: 50+ M(SD)* = 63(9)	Problem drinking (versus nonproblem drinking), as determined by the CAGE screener, was associated with higher risk of later anxiety and depression for women to a greater extent than for men during the 2-year followup. The findings were not significant when tested with the overall mixed sex sample.
Mejldal (2020)^91^	DSM-5 AUD typologies in relation to demographics and treatment outcomes	Y	Y	N	Nordic	122 (36%)	219 (64%)	Range: 60+ Median = 65	Three classes of AUD typology were defined among older men and women engaged in a clinical treatment trial for alcohol use. Women were more likely than men to belong to a class distinguished by symptoms of irresistible cravings, drinking more than intended, and continued use despite consequences.
Holdsworth (2017)^94^	Examination of baseline predictors and course of alcohol consumption among older adults over 10-year period	N	S	N	UK	2,635 (57%)	2,016 (43%)	Range: 50+ M = 62 at baseline	Higher income, more education, being employed, and smoking were associated with relatively more frequent drinking and more drinks on drinking days in women (measured as continuous variables). Women who had no partner or who had lost/separated from their partner (versus those with a romantic partner) had the steepest decline in drinking over 10 years. Deterioration in health over time was associated with decreases in the number of drinks per week compared to those with consistently poor health or improved health over time.
Dauber (2018)^95^	Analysis of data from adults in addiction treatment	Y	Y	N	Western Europe	3,771 (35%)	7,089 (65%)	Range: 60+ M = 65	Senior women had positive treatment outcomes. Compared to men, women were more likely to be widowed and living alone, differed in reasons for drinking, had higher retirement rates, and had later AUD onset. Frequent contacts with doctors could play an important role in detection of AUD and initiation of treatment.
Brennan (2011)^97^	Latent growth model of baseline predictors of alcohol use and alcohol-related problems over 20 years	Y	Y	Y	USA	320 (45%)	399 (56%)	Range: 55–65 M(SD)* = 61(3) at baseline	For men and women, social approval of alcohol use and reported use of substances for tension reduction correlated with more alcohol use and related consequences at baseline but were not associated with changes in drinking over 20-year follow-up for women. From baseline to 10-year follow-up, both men and women reported decreased alcohol-related problems, leveling off between 10- and 20-year follow-up. Baseline alcohol-related problems were associated with earlier decline in drinking among older women compared to men.
Bosque-Prous (2017)^101^	Cross-sectional study on individual and environmental correlates of hazardous drinking (defined by positive AUDIT-C screen)	N	S	Y	Multiple	35,892 (54%)	30,063 (46%)	Range: 50+ 49% 50–64 51% 65+	Screening positive on the AUDIT-C (≥ 4 for women, ≥ 5 for men) was associated with tobacco use among men and women. Women who perceived their health as poor to fair (compared to good to excellent) and who were divorced/widowed (compared to living with a spouse) were less likely to drink hazardously (for the latter, the opposite trend was found for men). Women, but not men, with upper secondary education (compared to less education) were more likely to screen positive on AUDIT-C.
Reczek (2016)^102^	Mixed-methods study examining marital status and alcohol use over 18 years	N	Y	Y	USA	6,058 (60%)	4,003 (40%)	M(SD) = 61(9) at baseline	Being married, including remarried, increased likelihood of women engaging in heavy drinking (≥ 3 drinks at least 1 day/week in past 3 months) versus being never married or previously married. The opposite was found for men. Divorced women who drank decreased their drinking more quickly as they aged, compared with stably married women who drank.
Christie (2013)^186^	Retrospective analysis of 20 years of data from older adults entering alcohol treatment	Y	Y	N	Canada; UK	225 (39%)	360 (61%)	Range: 60+ M* = 66	Profile of the typical female age 60+ at assessment for alcohol treatment was, on average, a 67-year-old woman with daily wine or spirit consumption who drank at home alone for reasons of anxiety or loneliness/depression.
Moos (2010)^187^	Survey of health and alcohol use, completed by adult participants three times over 20 years	Y	N	N	USA	320 (45%)	399 (55%)	Range: 55–65 at baseline M = 61	An association was found over time between a reliance on alcohol to manage pain and an increasing amount of alcohol consumption in both women and men.
Lemke (2008)^188^	Cross-sectional survey on correlates of alcohol-related problems in older adults	Y	Y	N	USA	347 (42%)	484 (58%)	Range: 62–78 M* = 69	Women reporting alcohol-related problems had more exposure to stressors (partner drinking, family problems, death of someone close, emotional distress) than men with alcohol-related problems but did not differ from women without alcohol-related problems. Women with alcohol-related problems were more likely to drink in response to stress than women without such problems.
***Unique Treatment Needs—Use and Misuse of Prescribed Medications and Cannabis***									
Al-Rousan (2015)^58^	Cross-sectional correlates with binge drinking in a sample age 65+	N	N	N	USA	10,328 (55%)	8,466 (45%)	Range: 65+ M = 76	Compared to women who did not binge drink in the past month, those who did binge drink had a lower prevalence of chronic disease, higher education, and higher prevalence of tobacco or cannabis use.
Stevenson (2005)^63^	Cross-sectional study on predictors of alcohol use among older women	N	N	N	USA	135 (100%)	0 (0%)	Range: 60+ M = 69	“Best predictor model” of alcohol use included regular use of > 1 over-the-counter medications, heavier coffee consumption, and using alcohol to fall asleep.
Blazer (2009)^99^	Secondary, cross-sectional data analysis of a national survey on alcohol use and binge drinking in adults age 50+	N	S	Y	USA	6,001 (55%)	4,952 (45%)	Range: 50+	For men and women, binge drinking (defined as ≥ 5 drinks) was associated with tobacco and illicit drug use compared to not drinking (not accounting for former drinking). Among women only, nonprescribed drug use was associated with binge drinking; marital status was associated with drinking patterns in men but not women. Results varied when binge drinking was compared to not drinking versus other patterns of alcohol use, for men and women.
Tevik (2017)^189^	Cross-sectional study examining alcohol and drug use	Y	Y	N	Nordic	6,084 (53%)	5,461 (47%)	Range: 65–101 Median = 73	Compared to their male counterparts, women who reported ≥ 1 drinking days per week were more likely to use drugs with addiction potential (benzodiazepines, nonbenzodiazepine hypnotics, or opioids).
Serdarevic (2019)^190^	Analysis of clinical correlates among older women with or without binge drinking, with and without prescription opiate use	N	N	N	USA	2,370 (100%)	0 (0%)	Range: 50+ M(SD) = 61(8)	In this sample (53% Black; 46% food insecure; 27% employed), 30% of women used prescription opioids and/or binge drank in the past 30 days. Women who both used prescription opioids and binge drank in the past 30 days were more likely to report back pain (71%) than those who binge drank only or who engaged in neither. Concurrent or separate prescription opioid and binge drinking in the past 30 days were associated with comorbid depression and anxiety.
Barnes (2010)^191^	Cross-sectional survey data collected as part of a larger trial of an educational intervention about at-risk drinking	Y	Y	N	USA	1,594 (48%)	1,714 (52%)	Range*: 60+	People with “at-risk” drinking were identified using the CARET risk evaluation tool based on alcohol-related behavior, including consuming alcohol in combination with medical comorbidities worsened by alcohol, or with alcohol-interactive medications. 25% of women age 60+ in primary care clinics were engaging in these types of drinking behaviors.
***Unique Treatment Needs—Subpopulations***									
Bryan (2017)^104^	Survey about heavy drinking in lesbian, gay, and bisexual women and men	N	S	Y	USA	1,081 (46%)	1,270 (54%)	Range*: 50–98 M(SD)* = 61(8)	Among lesbian and bisexual older women, current smoking, younger age, income > 200% of the federal poverty level, not being in recovery from alcohol or drugs, greater social support, and lower perceived stress were all associated with increased likelihood of heavy drinking compared to no drinking.
Serdarevic (2019)^190^	Analysis of clinical correlates among older women with or without binge drinking, with and without prescription opiate use	N	N	N	USA	2,370 (100%)	0 (0%)	Range: 50+ M(SD) = 61(8)	In this sample (53% Black; 46% food insecure; 27% employed), 30% of women used prescription opioids and/or binge drank in the past 30 days. Women who both used prescription opioids and binge drank in the past 30 days were more likely to report back pain (71%) than those who binge drank only or who engaged in neither. Concurrent or separate prescription opioid and binge drinking in the past 30 days were associated with comorbid depression and anxiety.
Zapolski (2017)^193^	Cross-sectional study examining racial/ethnic identity and binge drinking among women	N	S	Y	USA	106,517 (52%)	98,681 (48%)	Range*: 12+ 7% 50–64 4% 65+	Among those with income < $20,000, African American women ages 50–64 were almost twice as likely to have consumed ≥ 5 drinks on a single occasion over the past 30 days, compared to their White counterparts. At all income levels ≥ $20,000, African American and Hispanic women, each compared to White women of this age group, did not differ in the likelihood of drinking ≥ 5 drinks in the past 30 days.
Parsons (2014)^194^	Latent class analysis identified baseline patterns of substance use among HIV+ adults	N	Y	N	USA	175 (31%)	380 (68%)	Range*: 50–72 M(SD)* = 55(4)	Among substance users, four latent classes of substance use were identified: Exclusive Alcohol Use, Alcohol and Marijuana Use, Alcohol and Cocaine/Crack Use, and Multiple-Substance Use. Heterosexual women were disproportionally over-represented in the No Use and Exclusive Alcohol Use classes and under-represented in the Multiple-Substance Use class. The proportion of lesbian women did not differ across classes; this could be due to the low number in the sample.
Rowan (2017)^195^	Qualitative study of factors that helped lesbian women quit drinking	Y	N	N	USA	20 (100%)	0 (0%)	Range: 50–70 M = 58	Five key factors helped participants attain and maintain sobriety: wake-up calls, formal treatment, 12-step recovery groups, consequences from other sources, and resiliency.

‡ Participants’ age range and mean (standard deviation), if provided. Values for female participants only are provided when available.

* Statistics provided only for the combined male and female sample.

a **Quitters:** “Y” indicates that the study accounted for participants who had a history of heavy alcohol use or AUD, accounting for possible “sick quitter” effects in their analyses. Articles either (1) assessed for past drinking habits or symptoms of AUD and then accounted for this data in their statistical analyses or (2) limited their sample to current alcohol users only. “N” indicates that the study did not assess for past alcohol use/AUD or account for it in analyses.

b **Sex Differences:** “Y” indicates that male and female participants were directly compared in analyses. “N” indicates that they were not compared. “S” indicates studies that ran stratified or otherwise separated analyses for male and female participants.

c **Age Differences:** “Y” indicates that age was tested in relation to outcomes, either as a continuous or categorical (e.g., comparing 60–69 to 70–79, etc.) variable; “N” indicates that the full sample was ≥ 50 years old and no age analyses were conducted.

*Note:* AUD, alcohol use disorder; AUDIT, Alcohol Use Disorders Identification Test; AUDIT-C, Alcohol Use Disorders Identification Test–Concise; BMI, body mass index; CARET, Comorbidity Alcohol Risk Evaluation Tool; DSM, *Diagnostic and Statistical Manual of Mental Disorders*^29^; ICD, *International Classification of Diseases*^28^; SMAST-G, Short Michigan Alcoholism Screening Test-Geriatric Version.

**Appendix 4:** Extraction Table of Reviewed Articles Related to Treatment and Prevention of Alcohol Misuse in Older Women

Author	Study Method	Quitters a	Sex Differences^b^	Age Differences c	Country	Total *n*, % (Female)	Total *n*, % (Male)	Participant Age (Years)‡	Summary of Findings
***Older Women in Sex-Neutral Treatment***									
Satre (2004)^197^	Comparison of 5-year treatment outcomes of adults ages 55–77, 40–54, and 18–39 in treatment	NA	Y	N	USA	17 (26%)	48 (74%)	Range: 18–77	Older women (ages 55–77) were more likely to be abstinent at 5 years after treatment than older men and younger women (ages 18–39). Older women stayed in treatment longer (23 weeks) than older men (10 weeks).
Satre (2007)^198^	Secondary data analysis from RCT testing treatment intensity in an outpatient setting	NA	Y	N	USA	25 (30%)	59 (70%)	Range: 55–77 M(SD): 60(5) at baseline	At 7 years after treatment, women had better long-term outcomes than men; 76% of women reported total abstinence versus 54% of men. Women’s number of days in treatment (143 days) exceeded that of men (80 days); across sex, number of days in treatment predicted better outcome.
Satre (2012)^199^	Secondary data analysis of RCT comparing levels of treatment intensity in an outpatient treatment	NA	Y	Y	USA	620 (38%)	1,026 (62%)	Compared three age groups: at baseline, ages 18–39, 40–54, 55+	Across 5-, 7-, and 9-year follow-up, adults ages 40–54 or age 55+ were more likely than those ages 18–39 to be in remission and abstinent. Female sex was associated with remission across years 5–9. Results highlight relatively favorable long-term prognosis of older women in AUD treatment. Other factors associated with remission in years 5–9 were not losing a partner; no decline in health; close friends supportive of recovery; and no close friends who encouraged alcohol use. Negative life transitions (e.g., losing a partner, decline in health) were associated with worse outcomes.
Kuerbis (2017)^200^	RCT testing feasibility of online normative feedback versus personalized feedback for drinkers age 50+	NA	Y	N	USA	63 (46%)	75 (54%)	Range: 50+	With no sex differences, 80% of participants rated themselves as drinking at “no or low-risk” levels, yet 52% were found to be drinking at “at-risk” levels. Feedback was helpful, resulting in 44% of recipients planning to change drinking. Results favored the normative feedback condition. Participants preferred online (41%) to a brief in-person (32%) intervention.
Andersen (2020)^201^	RCT testing motivational enhancement therapy (MET) + community reinforcement for senior approach to MET only	NA	Y	N	USA; Western Europe; Nordic	279 (40%)	414 (60%)	Range: 60+ Median* = 64	About 50% of participants in each study condition met criteria for “treatment success” (i.e., having a blood alcohol content ≤ 0.05 in the 30 days before assessment) at 26 weeks follow-up. Women were less likely than men to meet a threshold of treatment success. No conclusions about efficacy for women separate from men could be made in overall alcohol use, but both treatments appeared promising for both sexes.
Tryggedsson (2023)^202^	Secondary data analysis from the parent RCT (Andersen et al.) described above	NA	Y	N	USA; Western Europe; Nordic	279 (40%)	414 (60%)	Range*: 60+ Median: 65	Women and men age 60+ had improved quality of life after both 4 and 12 weeks of treatment, with improvements lasting at least 1 year. Women had fewer alcohol-free days in the year following treatment than men.
Seddon (2023)^203^	Secondary analysis comparing men and women with very late-onset (age 60+) problem drinking at entry and follow up	NA	Y	N	UK	344 (44%)	436 (56%)	Range*: 50–88 M* = 60	The intervention tested (Drink Wise Age Well) provided age-sensitive screening, support groups, and interventions adapted for older adults age 50+ with alcohol problems. Self-reported age of “problem drinking” onset was unrelated to sex or treatment outcome. Overall, women decreased drinking after completing treatment more than men.
Satre (2004)^204^	Secondary data analysis from RCT of treatment intensities with sex-difference analyses	NA	Y	N	USA	29 (32%)	63 (68%)	Range: 55–77 M(SD) = 60(5)	At 6-month follow-up, women reported higher rates of abstinence in prior 30 days compared to men. Longer stay in treatment for both sexes predicted abstinence at 6 months.
***Older Women in Age-Neutral Female-Specific Treatment***									
Gjestad (2011)^205^	RCT for women with AUD testing early treatment for women with addiction (EWA) compared to mixed-sex treatment-as-usual condition	NA	NA	Y	Nordic	200 (100%)	0 (0%)	Range: 71+ at 27-year follow-up M(SD) = 42(10) at baseline	EWA treatment consisted of detoxification, inpatient treatment as needed, psychotropic medications and/or disulfiram, individual and women-only group therapy two to three times per week, regular staff contact for 2 years post baseline, work training, physiotherapy, family therapy, and partner involvement. For women (including older women), reduced mortality was found in the EWA group compared to the mixed-sex treatment-as-usual condition.
Al-Otaiba (2012)^206^	Secondary data analysis from RCT comparing three age categories among alcohol-dependent females in response to sex-neutral or female-specific CBT/MET	NA	NA	Y	USA	181 (100%)	0 (0%)	Range: 25–69 M (age 55+) = 62 M (ages 45–55) = 49 M (age < 45) = 38	At baseline, women age 55+ had better psychosocial functioning, more supportive social networks, less severe substance use history, and more alcohol use compared to younger age groups. At the end of treatment, older women attended more sessions, completed more homework, and showed greater reductions of drinking frequency and percentage of heavy drinking days related to the younger age groups. Main effects were found for improvement in depression, anxiety, autonomy, and coping skills. Women age 55+ engaged well and reported positive drinking and other “recovery” outcomes during and up to 12 months after CBT/MET for AUD.
***Untreated Remission***									
Schutte (2006)^208^	Secondary analysis of a 10-year study comparing untreated versus treated remission cases	NA	Y	N	USA	226 (39%)	352 (61%)	Range: 55–65	Untreated women were more likely to achieve remission of alcohol use than untreated men (46% versus 28%, respectively). Women who were advised to reduce drinking attained remission more often and were more responsive to such advice than men.
***Pharmacological Interventions***									
Oslin (2005)^209^	RCT examining efficacy of sertraline + compliance therapy, with or without naltrexone, on alcohol use and depression	NA	Y	N	USA	15 (20%)	59 (80%)	Range: 55+ M* = 63	72% of women treated with sertraline + psychosocial support had and “overall response to treatment” (response in both abstinence and depression remission), compared to 25% of women who were treated with sertraline + psychosocial support plus naltrexone; men showed no outcome difference by condition.
***Prevention***									
Laberge (2021)^57^	Cross-sectional study of associations between health and heavy drinking in older adults	N	N	N	Canada	1,324 (58%)	950 (42%)	Range*: 65+ M(SD) = 73(6)	Compared to women who did not drink, those who consumed ≥ 1 drink/week were less likely to self-rate physical health as poor. Women who did not drinks were more likely than those who did to be ill or disabled before retirement. Women consuming ≥ 7 drinks/week were more likely to be living with a spouse or partner, compared to those who did not drink or those who consumed one to two drinks/week.
Balsa (2008)^60^	Cross-sectional associations of alcohol and health among older adults	Y	N	N	USA	2,587 (38%)	4,274 (62%)	Range: 65+ M = 76	Any alcohol consumption up to 2 drinks/day by women was associated with better self-perceived health status and with lower use of health services; this association of drinking and health was diminished with increasing alcohol use.
Stelander (2022)^61^	Cross-sectional study examining correlates of at-risk drinking per AUDIT	Y	N	N	Nordic	4,451 (52%)	4,165 (48%)	Range: 60+ 60% 60–69 31% 70–79 9% 80+	Among women being at risk of AUD (AUDIT-C score ≥ 3) and problem drinking (per AUDIT individual items) were associated with lower age and higher education. Better self-reported health, living with a spouse/partner, and more social support were associated with greater likelihood of at-risk drinking, but lower likelihood of reporting problems/consequences related to drinking.Mental distress and the use of sleeping pills were associated with higher likelihood of at least one alcohol-related problem.
Holdsworth (2017)^94^	Examination of baseline predictors and course of alcohol consumption among older adults over 10-year period	N	S	N	UK	2,635 (57%)	2,016 (43%)	Range: 50+ M = 62 at baseline	Higher income, more education, being employed, and smoking were associated with relatively more frequent drinking and more drinks on drinking days in women (measured as continuous variables). Women who had no partner or who had lost/separated from their partner (versus those with a romantic partner) had the steepest decline in drinking over 10 years. Deterioration in health over time was associated with decreases in the number of drinks per week compared to those with consistently poor health or improved health over time.
Holton (2019)^96^	Longitudinal study on alcohol use, health and retirement	N	S	N	Ireland	2,353 (55%)	1942 (45%)	Range: 50+ M = 62 at baseline	Women with self-reported poor versus good health drank less at baseline. Increased drinking frequency over time was associated with higher education and tobacco use in men and women. Binge drinking (defined as 4.3 U.S. standard drinks/occasion) was less frequent among rural versus urban women. Those who did not drink and had lower education and fair health were most likely to be lost to attrition, potentially skewing results.
Bosque-Prous (2017)^101^	Cross-sectional study on individual and environmental correlates of hazardous drinking (defined by positive AUDIT-C screen)	N	S	Y	Multiple	35,892 (54%)	30,063 (46%)	Range: 50+ 49% 50–64 51% 65+	Screening positive on the AUDIT-C (≥ 4 for women, ≥ 5 for men) was associated with tobacco use among men and women. Women who perceived their health as poor to fair (compared to good to excellent) and who were divorced/widowed (compared to living with a spouse) were less likely to drink hazardously (for the latter, the opposite trend was found for men). Women, but not men, with upper secondary education (compared to less education) were more likely to screen positive on AUDIT-C.
Towers (2018)^108^	Secondary data analysis of a longitudinal survey that looked at correlates of alcohol use	Y	S	Y	New Zealand	1,529 (52%)	1,399 (48%)	Range: 52–86 M(SD) = 66(8)	For both men and women over age 50, the association between drinks per day and general physical health (self-reported functional health and well-being) followed the same trends as the association between drinks per day and socioeconomic status (SES), suggesting confounding covariance between SES and alcohol use in relation to health. The association of drinking and health was substantially reduced after controlling for a direct measure of SES.
Davies (2024)^136^	Eight focus groups conducted with 30 women and six expert stakeholders to develop a prevention intervention	NA	N	N	UK	35 (97%)	1 (3%)	Range: 40–65 M = 50	Three major themes were identified by participants as useful: understanding ineffective messaging, transitions and challenges, and message acceptability. Current health information about alcohol was perceived as judgmental, and breast cancer awareness was put down to chance. Mid/older life was associated with challenges that could lead to increased consumption. The menopause transition was identified as a possible key moment for alcohol reduction. The authors highlighted key barriers and enablers to communicating risk information and encouraging alcohol reduction.
Chapman (2020)^185^	Secondary analysis of data on knowledge of alcohol use; the National Health and Medical Research Council in Australia stipulates that adults (men and women) should drink ≤ 2 drinks/day (1.4 U.S. standard drinks) and < 4 drinks (~3 U.S. drinks) on a single occasion	NA	Y	N	Australia	6,181 (52%)	5,705 (48%)	Range: 50+	More females than males did not know the definition of low-risk drinking for short- or long-term consequences (as defined in Australia, see left). Women overestimated the number of drinks that equate with Australia’s guidelines for alcohol use, estimating that 3.3 drinks/day were withing guidelines. Among women with heavy drinking, 33% overestimated the number of drinks meeting heavy drinking guidelines and 48% overestimated the number of drinks qualifying for nonheavy drinking. Among women who drank heavily and accurately estimated guidelines, only 43% perceived any harm related to their drinking.
Mauro (2021)^211^	Secondary analysis of data from participants with past-year alcohol use and health care use	NA	Y	N	USA	4,946 (51%)	4,717 (49%)	Range: 65+	More than a quarter of older adults who used alcohol reported that they were not asked about their drinking by their healthcare providers. Older women were less likely than older men to report having any discussion about alcohol use with health care providers.
Wilkinson (2016)^212^	Survey of older Australian individuals who were drinking on knowledge about alcohol guidelines and acceptability of general practitioner screening	NA	Y	N	Australia	94 (50%)	94 (50%)	Range: 60–89 M = 69	90% of women were receptive to their general practitioner asking about their alcohol use; only 20% of women recalled their general practitioner raising this issue. Regarding health risks, 68% of women believed that spirit-based drinks increased health risks, 60% thought that red wine decreased health risks, and 22% believed red wine increased health risks. Although 60% of women said they knew the guidelines for women, only 44% of those 60% accurately identified them.
Grigg (2023)^213^	RCT of brief alcohol intervention (“Health4Her”) + lifestyle health prevention versus prevention only for women at breast cancer screening	NA	N	N	Australia	557 (100%)	0 (0%)	Range: 40–87 M = 60	Awareness that alcohol use increases risk of breast cancer was higher at follow-up for both interventions but favored the “Health4Her” arm. Alcohol literacy also increased more in the “Health4Her” condition, as did secondary outcomes.
Tivis (2008)^214^	Survey and physical exam to assess drinking in relation to alcohol use and measures of general health functioning	Y	N	N	USA	115 (100%)	0 (0%)	Range: 50–65 M = 57	Women’s (ages 50–65) decisions about drinking seemed to be made within a larger lifestyle context that included decisions about diet and exercise. Women who drank above recommended levels, compared to those who did not drink or had other levels of drinking, were at increased risk for cardiovascular problems. The “nondrinker” group was confirmed not to have any history of alcohol-related problems or AUD.
Moos (2005)^215^	Prospective study, with survey data and 10 years of follow-up	NA	Y	N	USA	529 (41%)	762 (59%)	Range*: 53–68 M(SD)* = 61(3)	Four years post-baseline, women who had a greater health burden (versus less health burden) were more likely to be abstinent from alcohol.
Oliveira (2019)^216^	Survey of adults to identify guidelines for alcohol use to reduce dementia risk	NA	Y	N	UK	2,880 (73%)	1,060 (23%)	Range: 50+ M* = 62	74% of the mixed sex sample who drank were likely to follow the “low-risk” drinking guidelines if it meant their risk of developing dementia could be reduced. Women were more likely than men to follow alcohol guidelines.

‡ Participants’ age range and mean (standard deviation), if provided. Values for female participants only are provided when available.

* Statistics provided only for the combined male and female sample.

a **Quitters:** “Y” indicates that the study accounted for participants who had a history of heavy alcohol use or AUD, accounting for possible “sick quitter” effects in their analyses. Articles either (1) assessed for past drinking habits or symptoms of AUD and then accounted for this data in their statistical analyses or (2) limited their sample to current alcohol users only. “N” indicates that the study did not assess for past alcohol use/AUD or account for it in analyses. “NA”, used in treatment and prevention studies, reflects that accounting for sick quitters was not relevant because either they were all seeking treatment for AUD (in treatment studies) or did not necessarily need to have any alcohol-related problems to be part of the study (in prevention studies, if not related to the outcome measure, such as screening rates).

b **Sex Differences:** “Y” indicates that male and female participants were directly compared in analyses. “N” indicates that they were not compared. “S” indicates studies that ran stratified or otherwise separated analyses for male and female participants. “NA” indicates that analysis of sex differences was not applicable because the study population only included women.

c **Age Differences:** “Y” indicates that age was tested in relation to outcomes, either as a continuous or categorical (e.g., comparing 60–69 to 70–79, etc.) variable; “N” indicates that the full sample was ≥ 50 years old and no age analyses were conducted.

*Note:* AUD, alcohol use disorder; CBT, cognitive behavioral therapy; MET, motivational enhancement therapy; RCT, randomized controlled trial; SES, socioeconomic status.
